# Atomistic Origins of Conductance Switching in an ε-Cu_0.9_V_2_O_5_ Neuromorphic Single Crystal Oscillator

**DOI:** 10.1021/jacs.4c11968

**Published:** 2024-12-04

**Authors:** John Ponis, Nicholas Jerla, George Agbeworvi, Saul Perez-Beltran, Nitin Kumar, Kenna Ashen, Jialu Li, Edrick Wang, Michelle A. Smeaton, Fatme Jardali, Sarbajeet Chakraborty, Patrick J. Shamberger, Katherine L. Jungjohann, Conan Weiland, Cherno Jaye, Lu Ma, Daniel Fischer, Jinghua Guo, G. Sambandamurthy, Xiaofeng Qian, Sarbajit Banerjee

**Affiliations:** 1 Department of Chemistry, Texas A&M University, College Station, Texas 77843, United States; 2 Department of Physics, University at Buffalo, State University of New York, Buffalo, New York 14260, United States; 3 Department of Material Science and Engineering, Texas A&M University, College Station, Texas 77843, United States; 4 Advanced Light Source, Lawrence Berkeley National Laboratory, Berkeley, California 94720, United States; 5 National Renewable Energy Laboratory, Golden, Colorado 80401, United States; 6 Material Measurement Laboratory, National Institute of Standards and Technology, Gaithersburg, Maryland 20899, United States; 7 National Synchrotron Light Source II, Brookhaven National Laboratory, Upton, New York 11973, United States

## Abstract

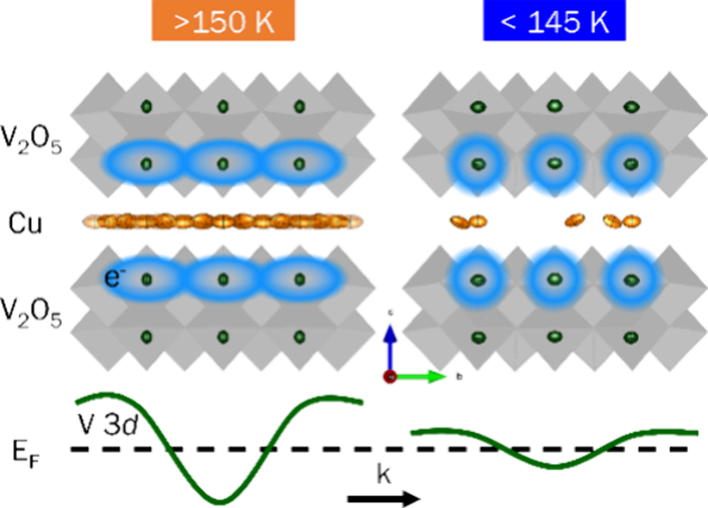

Building artificial neurons and synapses is key to achieving the promise of energy efficiency and acceleration envisioned for brain-inspired information processing. Emulating the spiking behavior of biological neurons in physical materials requires precise programming of conductance nonlinearities. Strong correlated solid-state compounds exhibit pronounced nonlinearities such as metal–insulator transitions arising from dynamic electron–electron and electron–lattice interactions. However, a detailed understanding of atomic rearrangements and their implications for electronic structure remains obscure. In this work, we unveil discontinuous conductance switching from an antiferromagnetic insulator to a paramagnetic metal in ε-Cu_0.9_V_2_O_5_. Distinctively, fashioning nonlinear dynamical oscillators from entire millimeter-sized crystals allows us to map the structural transformations underpinning conductance switching at an atomistic scale using single-crystal X-ray diffraction. We observe superlattice ordering of Cu ions between [V_4_O_10_] layers at low temperatures, a direct result of interchain Cu-ion migration and intrachain reorganization. The resulting charge and spin ordering along the vanadium oxide framework stabilizes an insulating state. Using X-ray absorption and emission spectroscopies, assigned with the aid of electronic structure calculations and measurements of partially and completely decuprated samples, we find that Cu 3*d* and V 3*d* orbitals are closely overlapped near the Fermi level. The filling and overlap of these states, specifically the narrowing/broadening of V 3*d*_*xy*_ states near the Fermi level, mediate conductance switching upon Cu-ion rearrangement. Understanding the mechanisms of conductance nonlinearities in terms of ion motion along specific trajectories can enable the atomistic design of neuromorphic active elements through strategies such as cointercalation and site-selective modification.

## Introduction

Conventional computing architectures based on complementary metal oxide semiconductor (CMOS) devices are constrained in speed and limits of energy consumption by fundamental thermodynamic limitations of charge-transfer processes in electrostatically modulated semiconductor thin films, and by the von Neumann bottleneck of having to shuttle data between separate logic and memory units.^1−4^ Nonvon-Neumann devices that thread together and logic and memory within a singular fabric have attracted much recent attention for energy efficient computing with the overarching goal of emulating information processing modalities of the human brain.^4−7^ Unlike CMOS architectures where the room-temperature Fermi—Dirac distribution of electron energies limits the steepness of the subthreshold voltage swing to a 60 mV/decade increase in current across a transistor channel, the strongly nonlinear dynamical response of memristive architectures holds promise for altogether different computing primitives reminiscent of synaptic and neuronal functionality in the human brain. Such computing primitives afford unprecedented efficiencies in terms of transistor count, time to solution, and energy per spike, and are particularly well suited to the needs of neural-network and oscillator-based computation, which are pivotal to enabling and accelerating artificial intelligence^7−9^ at scale.^10^

Since the advent of the memristor and demonstration of spiking neural networks in physical systems,^11,12^ several material archetypes have been explored as active elements for neuronal and synaptic elements. Electro-thermal nonlinearities are derived from diverse mechanisms ranging from filament formation to vacancy channel formation, diffusive dynamics of ion diffusion, and metal—insulator transitions.^5,8,13,14^ As a notable example, continuous regulation of ferroelectric polarization underpins high-fidelity synaptic emulation and has received much recent attention.^15−18^

Metal–insulator transitions, mediated by Mott and Peierls transitions in strongly correlated materials, show particular promise as active elements of energy efficient (fs/spike) and ultrafast (sub-ns) spiking circuits and oscillators.^11,19^ Much interest has focused on the identification of novel conductance switching mechanisms for neuromorphic computing but elucidation of design principles is stymied by incomplete knowledge of correlated changes in atomistic and electronic structure underpinning the nonlinear dynamical response. In this work, we build neuromorphic oscillators from single crystals and map the atomistic and electronic structure origins of conductance switching through high-resolution structure solutions across single-crystal transformations.

The vanadium oxide bronzes, with the general composition M_*x*_V_2_O_5_ where V_2_O_5_ crystallizes in a variety of single-layered, double-layered, and tunnel-structured polymorphs and M represents s-, p-, and d-block cations, exhibit a rich diversity of electronic phases and manifest a broad range of spin- and charge-ordering transitions. Such transitions are promising for neuromorphic computing since a highly nonlinear response is elicited with minimal entropy dissipation.^20^ These compounds comprise open V_2_O_5_ frameworks with guest ions arrayed along one-dimensional tunnels^21,22^ or within two-dimensional^23−25^ interstices, formally reducing adjacent vanadium centers from V^5+^ to V^4+^. The ionic radii, formal valence, and orbital configurations of the intercalated ions strongly modulate the atomistic and electronic structure of the V_2_O_5_ framework.^22^ This rich interplay of spin, atomic, charge, lattice, and orbital degrees of freedom between the guest ions and host lattice endow the vanadium oxide bronzes with highly articulated electronic structures, which are conducive to manifestation of nonlinear dynamical transformations desirable for neuromorphic computing.^8^ Understanding the mechanistic origins of these phenomena is necessary for tuning transformation characteristics (such as transition magnitudes, hystereses, and onset conditions) and to guide materials design.

The well-studied β-*M*_*x*_V_2_O_5_ tunnel-structured bronzes host a broad range of interstitial cations M. Conductance transitions in such materials are relatively insensitive to guest ion identity but sensitive to their stoichiometry, *x*, and occur independently of ion ordering,^21,26,27^ which implies that insulating states involve localization of electrons on the V_2_O_5_ framework, interacting only weakly with completely ionized interstitial ions. Among known tunnel bronzes, the cuprated member exhibits a uniquely complex electronic and structural phase diagram with conductivity transitions accompanied by Cu-ion ordering whose magnitude and commensurability depends closely on the copper concentration *x.*(28) The difference in behavior is ascribed to (i) preference of Cu ions for a distinctly small coordination site and tendency to split-site disorder, which enable relatively fast diffusion and (ii) the energetic proximity of V 3*d* and Cu 3*d* orbitals near the Fermi level, which renders the material’s electronic structure highly sensitive to copper ion order/disorder.^22^ Although less studied, the double-layer bronzes host a similarly diverse set of cations albeit in the interlayer space.^29^ The higher dimensionality of the interstices provides additional opportunities for positional correlation and long-range ordering of intercalated ions.

We report here temperature- and voltage-induced metal–insulator and paramagnetic—antiferromagnetic transitions in single crystals of a two-dimensional vanadium oxide bronze, ε-Cu_0.9_V_2_O_5_. Based on high-resolution single crystal structure solutions across the thermal transition, we uncover a distinctive superlattice ordering/melting of intercalated Cu ions between 2D V_4_O_10_ double layers that underpins charge and spin localization/delocalization. The short-range order/disorder of Cu ions mapped through single crystal diffraction and examined through molecular dynamics simulations, first-principles electronic structure calculations, magnetization measurements, and X-ray absorption and emission spectroscopies provides remarkable atomistic insight into the origins of nonlinear dynamical behavior critical for neuromorphic computing.

## Results and Discussion

### Neuromorphic Oscillators from Entire Single Crystals

**Figure 1:**
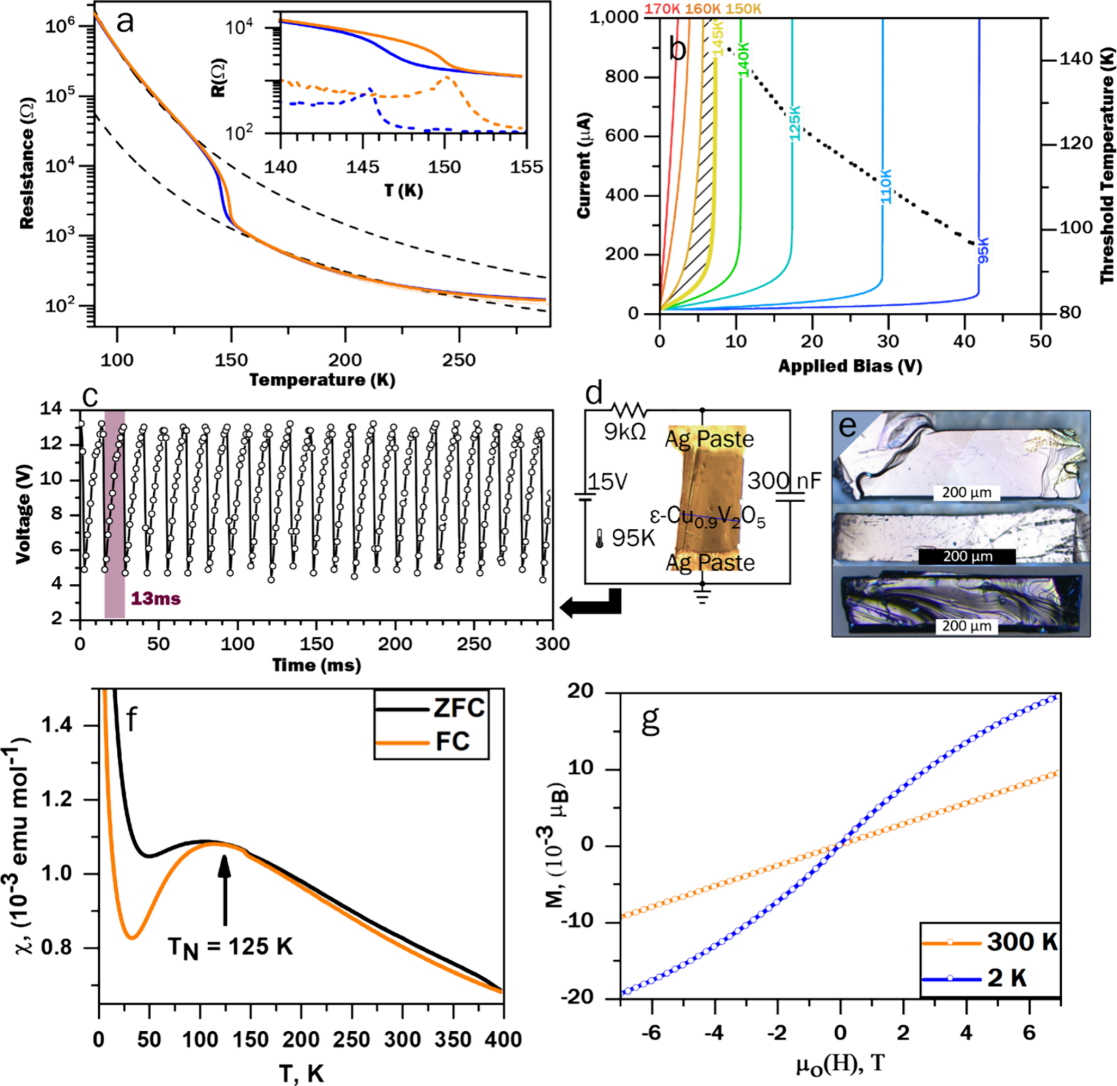
**Conductance nonlinearities, oscillatory behavior, and magnetic transitions in single-crystal ε-Cu**_**0.9**_**V**_**2**_**O**_**5.**_*(***a***) Resistance vs temperature, for a single crystal of* ε*-Cu*_*0.9*_*V*_*2*_*O*_*5*_*during cooling (blue) and heating (orange), with activated conduction fits (dashed lines) plotted above and below the conductivity transformation. The inset shows a detailed view of the conductance hysteresis (solid lines), with derivatives (dashed lines) indicating onsets at ca. 146 and 151 K during cooling and heating, respectively. (***b***) Current vs Voltage across a single crystal at several temperatures, demonstrating a voltage-induced reverse transition with a temperature-dependent critical voltage. The shaded region spans the first-order MIT. The dotted line shows the critical temperature of the voltage-induced transition. (***c***) Stable voltage oscillations measured across a single crystal oscillator, configured as depicted in (***d***). (***e***) Digital photographs of melt-grown* ε*-Cu*_*0.9*_*V*_*2*_*O*_*5*_*crystals. Crystal faces were indexed during single-crystal diffraction as described in the*Experimental Section. Layering along c* (facing the viewer) is visible as step-edges, whereas the longest crystal direction is oriented along the b-axis which displays the densest V – O bonding. (**f***) Temperature-dependent magnetic susceptibility of* ε*-Cu*_*0.9*_*V*_*2*_*O*_*5*_*powder, showing a clear Néel transition at T*_*N*_*= 125 K. (***g***) Magnetization vs magnetic field strength displaying sigmoidal shape characteristic of ferromagnetic ordering. As the temperature increases, the magnetization curve gradually becomes linear, indicating a reduction of the competing FM contribution.*

Single crystals of ε-Cu_0.9_V_2_O_5_ were prepared from powders by melt growth as described in the Experimental Section. Figure 1a shows the electrical conductivity of an individual millimeter-sized crystal, measured along the crystallographic *b-*axis, as a function of temperature. Single-domain crystals were selected for transport measurements and to be fashioned into oscillators based on careful indexing of single-crystal diffraction patterns. An abrupt, reversible hysteretic switching of conductance spanning almost 2 orders of magnitude is apparent at ca. 146 K (151 K) during cooling (heating). As depicted in Figure 1b, the current response is profoundly altered by this transition, developing a nonlinearity that deepens and forms a sharper “shoulder” as the temperature decreases, approximating an ideal switch at 95 K. This coupling of thermal and electrical driving forces of current—voltage nonlinearity provides the essential components of an electrothermal memristor. Conductance switching in several other single crystals is shown in Figure S1. As a first-order transition, it is inevitable that multiple domains are stabilized and give rise to the observed hysteresis. Despite inevitable variations in compositions, defect concentrations and carrier mobilities, the transition temperature and hysteresis are remarkably consistent across the single crystal devices suggesting at these dimensions the manifestation of the intrinsic physics of the material as modified by percolation of variously conducting domains. Insets to Figure S1 show variations in widths of differential plots; the differences in these effective hysteresis widths is likely a result of the different pinning of insulating (conductive) domains during temperature sweeps.^30,31^ The voltage-induced switching behavior suggests the utility of ε-Cu_0.9_V_2_O_5_ as the active element in an oscillator circuit; the time-varying output voltage of such a circuit measured at 95 K is shown in Figure 1c, with the corresponding circuit diagram and circuit parameters shown in Figure 1d. With a constant supply voltage of 15 V, a 300 nF capacitor placed in parallel with the crystal charges until the voltage across the crystal reaches the threshold value at which the crystal transitions to its high conductance state. At this point the capacitor discharges through the crystal until its voltage dips below the threshold for the reverse transition and the cycle repeats. Oscillatory behaviors such as demonstrated here can be exploited for computation in coupled-oscillator systems or neural networks.^1,9,32,33^ To the best of our knowledge, these represent the first macroscopic single crystals fashioned into oscillatory circuits (indeed, their macroscopic dimensions, highlighted in Figure 1e, underpin the relatively slow time constants), which enables high-resolution structure solutions across the electronic phases.

Magnetic susceptibility (χ) measurements are shown in Figure 1f and illustrate an antiferromagnetic (AFM) to paramagnetic behavior with a broad Néel transition (T_N_) at ≈125 K, essentially coincident with conductance switching (Figure 1a). This broad maximum observed around 125 K is characteristic of low-dimensional materials^34^ and is likely a result of isolated paramagnetic impurities.^35^ The low-temperature rise in susceptibility at 25 K is further likely a result of these isolated paramagnetic sites.^35,36^ The antiferromagnetic characteristics are ascribed to superexchange interactions sketched in Figure S2a considered with respect to the low-temperature ε-Cu_0.9_V_2_O_5_ structure described in subsequent sections. A significant divergence is observed between ZFC and FC curves at *T* < *T*_N_ and *T* > *T*_N_.

The 1/χ in the FC mode shown in Figure S2b is fit to the modified Curie–Weiss (CW) law (χ = χ_*0*_*+ C/(T−θ*)) and yields a Weiss constant of −130 K and effective magnetic moment, μ_eff_ = 1.74 μ_B_ consistent with the theoretical spin only value for V^4+^ (1.73 μ_B_, S = 1/2). The magnetization curve at 2 K shown in Figure 1g increases rapidly from 0 to ≈0.011 μ_B_ up to 4 T followed by a linear increase up to ≈0.02 μ_B_, which appears to be unsaturated up to 7 T indicating a tendency for AFM coupling. The curve also displays a characteristic feature of an S-shape, indicating the presence of a weak FM component. The complex magnetic behavior observed is ascribed to a competing AFM state with an intrinsic weak FM transition and explains the spin canting signature seen in the χ (T) plot. As the temperature increases (Figure 1f), the magnetization curve gradually becomes linear, indicating a reduction in the FM correlation contribution. The results thus indicate a transition from an antiferromagnetic insulator (low conductance state) to a paramagnetic metal (high conductance state) with increasing temperature.

### Oscillator Characterization

Oscillator circuits such as those described in Figure 1d constitute the primitive computing elements in coupled-oscillator neuromorphic architectures.^1,9,32,33^ In general, these circuits exploit the nonlinear dependence of active material electrical properties (typically resistance) on external fields (such as temperature, stress, or voltage) to couple circuit states to time-varying material state variables. When a system is equilibrated at a steady state adjacent to such a nonlinearity, at the so-called “edge of chaos”, small perturbations suffice to trigger large excursions from equilibrium such as neuron-like spiking or sustained oscillations. To incorporate such nonlinear dynamical elements into functional neuromorphic architectures requires understanding the boundaries of, and dynamics across, the edge of chaos region and how they can be tuned by adjusting active material properties and circuit parameters.

**Figure 2:**
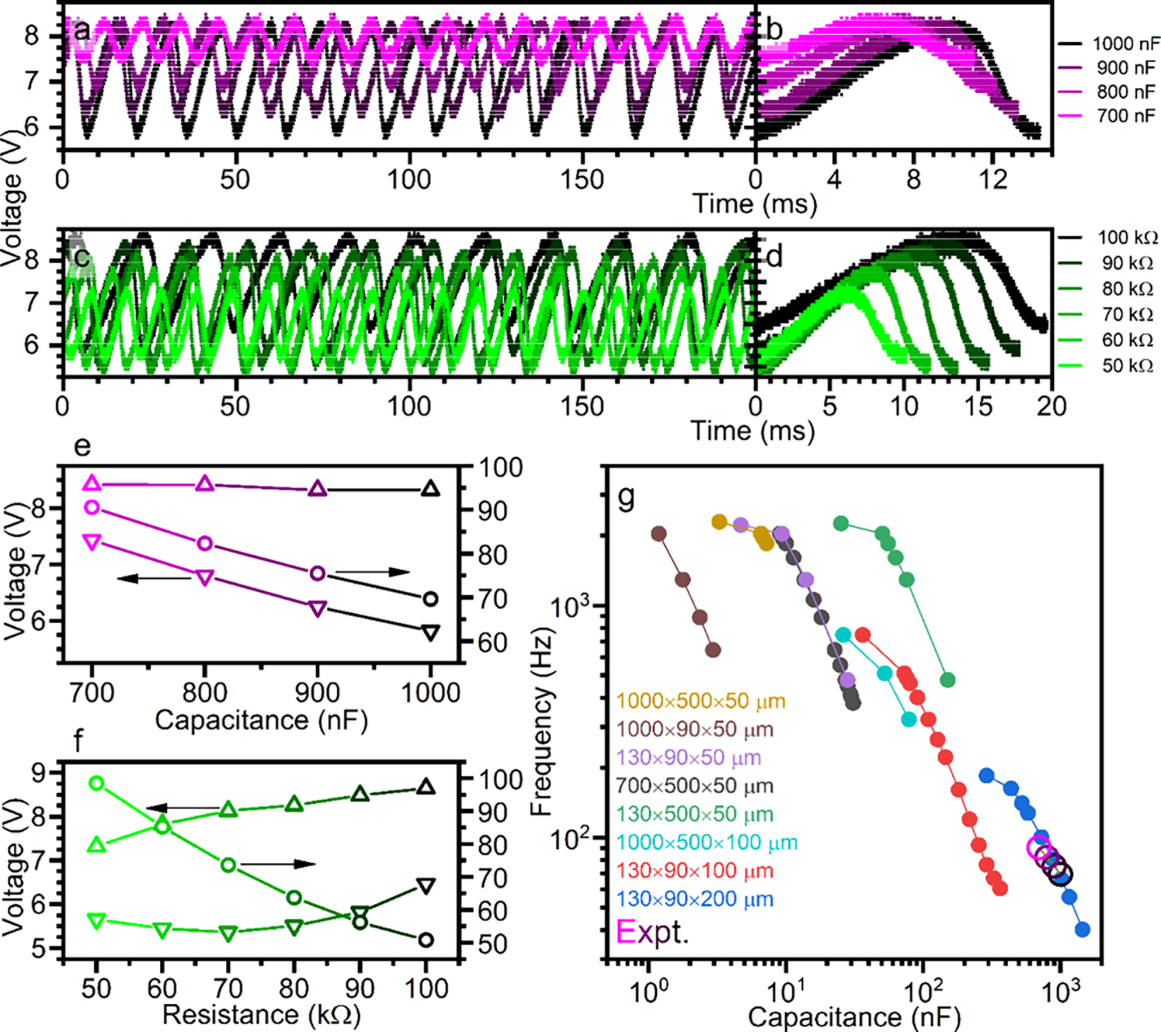
**Characterization of single crystal oscillators.** (**a-d**) *Oscillations exhibited by a single crystal of* ε*-Cu*_*0.9*_*V*_*2*_*O*_*5*_*with varying (***a***) capacitance and (***c***) resistance. Oscillator configured as shown in*Figure 1*d*, *with T = 95 K, V = 50 V, R*_*S*_*= 80 k*Ω*, C = 1000 nF, except where indicated otherwise. (***b,d***) show waveforms overlaid over a single oscillation period. (***e,f***) show respectively as functions of capacitance and resistance maximum (upward triangles) and minimum (downward triangles) voltage swings and frequencies (circles) of oscillations featured in (***a,c***). (***g***) Frequency vs total circuit capacitance predicted by an electrothermal oscillator simulation for crystals with indicated length × width × thickness and for the experimental results in (***e***)*.

Figure 2a and 2**c** show oscillations produced by the circuit in Figure 1d, with temperature *T* = 95 K, driving voltage *V* = 50 V with variations of series resistance and external parallel capacitance. Figure 2b and 2**d** overlay the corresponding waveforms over a single oscillation period. Measured values of frequency and minimum and maximum voltage are plotted vs the varied circuit parameter in Figure 2e and 2**f**. Additional details and discussion of oscillator characterization and behavior are provided in Figure S3.

In Figure 2a and 2**c**, a decrease in frequency is observed with increasing capacitance and resistance as expected, since both values contribute equivalently to the electrical time constant. The increase in voltage amplitude with capacitance implies a greater-than-linear increase in charge transferred through the crystal during each cycle. This is mainly driven by a corresponding decrease in the minimum voltage, suggesting that recovery of the insulating state is not simply triggered at a fixed threshold voltage but arises from additional oscillatory dynamics not captured by RC circuit physics. Motivated by these deviations from purely electrical behavior, we turn to an electro-thermal model for a quantitative understanding of the behavior of our ε-Cu_0.9_V_2_O_5_ oscillators.

Briefly, a circuit’s oscillation behavior can be parametrized by a dimensionless parameter **μ**, the ratio of the electrical and thermal time constants defined below in terms of the active material thermal resistance ***R*_t_**, total electrical resistance ***R*_e_**, active material thermal capacitance ***C*_t_**, and total electrical capacitance ***C*_e_**, all measured at the sink temperature ***T*_0_** and a characteristic voltage ***V*_0_**:^37^
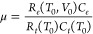
1

Figure S4a illustrates the oscillation voltage and temperature dependence upon μ and *R*_*e*_ predicted by the model. For values of μ less than a critical value **μ**_*c*_, thermal or electrical perturbations decay to a steady-state voltage and temperature as Joule heating and Newtonian cooling equilibrate. Increasing μ to μ_*c*_ (for instance, by adding an external capacitance to increase *C*_*e*_), the voltage and temperature responses undergo a Hopf bifurcation and stable oscillations occur upon perturbation. To the right of the bifurcation, μ/μ_*c*_ is effectively the phase offset between temperature and voltage fluctuations, and the oscillation frequency decreases as the waves further dephase at higher values of μ. The frequency versus capacitance curve in Figure 2e exhibits the inverse, nearly linear relationship predicted when μ is slightly above μ_*c*_, whereas the convergence toward zero oscillation amplitude with diminishing capacitance suggests that the Hopf bifurcation occurs at a capacitance ***C*_*min*_** between 500 nF and 600 nF.

Notably, the key motivation in this work is to use single-crystal-to-single-crystal transformations to obtain high-resolution single-crystal structure solutions to decipher the mechanistic basis for conductance nonlinearities (*vide infra*). However, the large size of the single crystal devices considered here are quite distinct from conventional nanometer-thick films. Crystal dimensions modify μ via several proportionality relationships, at first approximation:
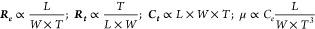
2where ***L*** is the length between electrical contacts, ***W*** is width perpendicular both to the conduction axis and substrate normal, and ***T*** is the thickness normal to the substrate. We expect then that μ is especially sensitive to crystal thickness, so that an *n*-fold increase in thickness requires a compensatory *n*^3^-fold increase in capacitance required to sustain oscillation behavior.

To further analyze the effects of crystal geometry on oscillator characteristics, we performed electro-thermal oscillator simulations as described in the Experimental Section. Frequency versus capacitance curves plotted in Figure 2g corroborate the geometric dependencies discussed above. Decreasing crystal width and especially thickness pursuant to device miniaturization will beneficially decrease the external capacitance required for oscillations, which favors high-aspect-ratio active elements. The simulated voltage and temperature oscillation amplitudes plotted in Figure S4b for selected crystal geometries reproduce the respective capacitance and μ dependences shown in Figure 2e and Figure S4a, capturing the interplay between Joule heating and Newtonian cooling. ε-Cu_0.9_V_2_O_5_ is an excellent candidate for exploring geometric effects on oscillator characteristics, as its layered nature allows for easy mechanical (tape) exfoliation, as shown in Figure S4**(c,d)**.

It is noteworthy that the region of stable oscillation is relatively narrow in this case given the first-order nature of the conductance nonlinearity and its relative abruptness.^37^ Such systems are prone to large unstable oscillations or attractions to distant steady states variant with the ratio of the thermal and electrical constants. For a given set of circuit parameters, oscillations in ε-Cu_0.9_V_2_O_5_ are stable only within a narrow temperature (and voltage) range, which is consistent with the highly nonlinear dependence of *C*_*t*_, *R*_*e*_, and *R*_*t*_ on *T*_*0*_, across the first-order temperature-induced structure and conductivity transition at (145 to 150 K). Figure S3d illustrates the nonmonotonic variation in frequency and amplitude exhibited across a narrow range of temperatures.

### Atomistic Mapping of Structural Transformations Underpinning Conductance Switching

**Figure 3:**
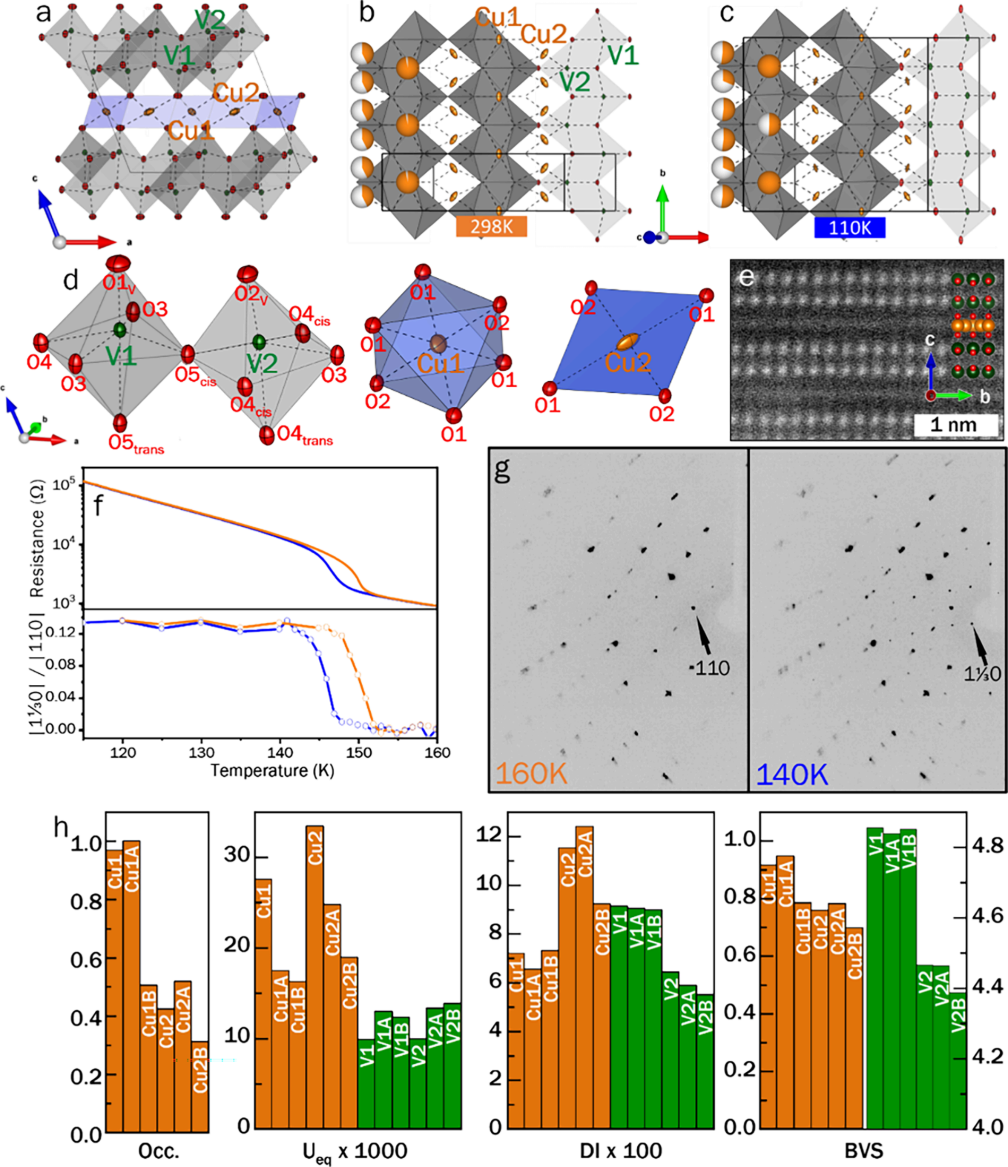
**Mapping structural origins of conductivity and magnetic transitions using single-crystal transformations.***(***a—c***) Crystal structure of* ε*-Cu*_*0.9*_*V*_*2*_*O*_*5*_*viewed down the crystallographic b-axis (***a**) and along the c*-axis at 298 K (**b***) and 110 K (***c***). The unit cell is demarcated in black. The Cu site occupancy is denoted by degree of circle filling. (***d***) Local coordination environments of vanadium atoms in the [V*_*4*_*O*_*10*_*] frameworks and Cu ions intercalated between [V*_*4*_*O*_*10*_*] layers. Cis and trans subscripts differentiate positions of space-group-equivalent oxygens relative to the vanadyl O*_*V.*_*(***e***) STEM image of a lamella cut from an* ε*-Cu*_*0.9*_*V*_*2*_*O*_*5*_*single crystal, with V (green), O (red) and Cu (orange) atoms highlighted. An expanded version of this image is found in*Figure S5g. *(***f***) Electrical resistance (upper panel) and 1**0 supercell reflection intensity, normalized to 110 reflection intensity, (bottom panel) versus temperature for a single crystal of* ε*-Cu*_*0.9*_*V*_*2*_*O*_*5*_*, during cooling (blue) and subsequent heating (orange) (see also*Video S1*). (***g***) Detector images taken above the transition temperature (left), showing only standard unit cell spots, and below the transition temperature (right), showing additional supercell reflections. (***h***) Structural descriptors for Cu (orange) and V (green) ions and coordination polyhedra. U*_*eq*_*is the equivalent isotropic thermal displacement; DI is the distortion index, the relative standard deviation of bond M-O bond lengths; and BVS is the Bond Valence Sum, the expected oxidation state calculated from M-O bond lengths.*^40^

To examine the structural origins of the conductivity and magnetization transition in ε-Cu_0.9_V_2_O_5_, we performed single-crystal X-ray diffraction experiments and obtained high-resolution structure solutions above and below the electronic transition. ε-Cu_0.9_V_2_O_5_ crystallizes in a 2D layered structural motif comprising chains of Cu1 and Cu2 ions alternating in the *a-*direction and arrayed along the *b-*direction, sandwiched between infinite [V_4_O_10_] double layers (Figure 3a). The double layers are constituted from [VO_6_] octahedra, which form edge-shared ladders along the *b-* axis, are connected by corner-shared oxygens along the *a-*axis and are dimerized by shared edges in the *c*-direction (Figure 3b).

Vanadium atoms are off-centered in their octahedral sites, forming a short “vanadyl” double-bond with the apical oxygen which projects into the interlayer space (O1_V_ and O2_V_ in Figure 3d). This vanadyl moiety has been implicated in mediating vanadium reduction by intercalated cations in analogous structures.^38,39^ STEM images (Figure 3e) taken along the *a*-axis of lamellae cut from single crystals corroborate the double-layered structure, and signs of layering are visible at several length scales in additional electron micrographs (Figure S5). Cu1 ions occupy distorted, edge-shared octahedral sites, with significant thermal displacement oriented along the *b* direction, whereas Cu2 ions occupy highly distorted square planar sites forming corrugated sheets along the *b* direction (Figure 3d), with principle thermal oscillation vectors forming a sinusoidal pattern along the *ab* plane. Tables S1—S4 respectively list structure solution information, atom positions, thermal displacement parameters, and bond distances and angles derived from structure solutions at 293 K.

Figures 3b and 3**c** contrast the 2D correlated superlattice ordering of Cu-ions between [V_4_O_10_] layers at low temperature and the considerable in-plane disorder manifested at higher temperatures. Figures S6a and S6b show residual Fourier density maps between Cu2 sites and Cu-ion ellipsoids fit to this residual density, respectively. The presence of this intersite Fourier density suggests thermally activated Cu site-hopping and a high degree of copper-ion mobility between the [V_4_O_10_] layers, particularly along the crystallographic *b* direction.^41^ The fundamental role of Cu-ion mobility and reordering in underpinning the electronic transition is evident in the high-resolution structure solutions delineating the underlying crystallographic transition, dynamical observations of the diminution of superlattice reflections with increasing temperature (Figure 3g and Video S1), and further validated by *ab initio* molecular dynamics simulations (*vide infra*).

At 293 K, the Cu1 sites are nearly completely occupied, whereas the Cu2 sites are approximately one-third filled. The short Cu2—Cu2 distance, 1.845 Å, precludes occupancy of adjacent sites; the observed occupancy thus represents roughly 2/3 of the maximum occupancy of the Cu2 site and allows for significant local disorder. Upon cooling below 145 K, coincident with the electronic transition, the crystal structure adopts a 1 × 3 × 1 supercell, derived from ordering of Cu ions between the [V_4_O_10_] layers. This supercell generates two equivalent sites, here labeled A and B, for each copper and vanadium site in the original unit cell. The occupancy of copper sites follows a high—high—low pattern along the chains in *b,* with the high occupancy in each case at the theoretical maximum of 1 and 0.5 for Cu1 and Cu2, respectively. It is notable that the distribution of copper content between chains changes, with migration of Cu ions from Cu1 to Cu2 upon transition to the insulating state, which reflects interchain Cu-ion mobility.

The relative intensity of the 10 supercell reflection measured during single-crystal diffraction experiments is plotted versus temperature in Figure 3f and is observed to be coincident with the conductivity transition, capturing even the 5 K hysteresis between heating and cooling transition temperatures. The diffraction patterns in Figure 3g demarcate the supercell reflections which appear below the transition temperature. Supporting Video S1 demonstrates the reversibility of the crystallographic transition, as the supercell spots first appear upon cooling, then disappear upon reheating. Notably, structural refinements of diffraction data collected from more than ten single crystals show almost identical patterns of Cu-ion ordering, differing only slightly in the magnitude of the occupancy fluctuation. Figure 3h illustrates several aspects of the structural transformation underpinning the electronic transition. The equivalent isotropic thermal displacement parameter (*U*_eq_), calculated from the three principle anisotropic thermal displacement vectors (see Table S3), decreases significantly for copper-ions in the insulating state. The increase in vanadium *U*_eq_ at low temperature is indicative of local variations in V-ion position due to “freezing-in” of Cu occupancy disorder. The Distortion Index (D.I.) is the mean relative bond length deviation, a measurement of the off-centering of a metal ion within in its coordination environment.^42^ In orthorhombic α-V_2_O_5_, the largest contribution to the D.I. comes from the distortion of the vanadium center toward the vanadyl oxygen, which constitutes a local coordination environment that is perhaps best described as a square pyramid rather than an octahedron. This bond has been shown to stiffen and decrease in length with increasing oxidation state of the central vanadium;^43^ the decrease in D.I. of V2 (particularly V2B) at low temperatures is thus indicative of increased electron localization on these vanadium sites.

Bond Valence Sum (BVS) values listed in Table S5(40) indicate partial charge disproportionation at Cu1, Cu2, and V2 sites at low temperature. Such charge localization stabilizes insulating states in low-dimensional transition metal oxides.^44^ The diffuse scattering and diffraction peak splitting visible in supporting Figure S7a and S7b suggest that the supercell described here is a manifestation of a more complex long-range structure modulation.^45^ The positional correlation between Cu ions in the c* direction transmitted through the intervening V_2_O_5_ sublattice layers by structural distortions (possibly accompanying electron localization) is further discussed in the Supporting Information. The diffraction experiments detailed above establish the connection between both inter- and intrachain Cu-ion mobility and superlattice ordering. We conclude from these results together with transport measurements that crystallographic transformations, observed here across entire single crystals, underpin conductance switching in ε-Cu_0.9_V_2_O_5_.

**Figure 4:**
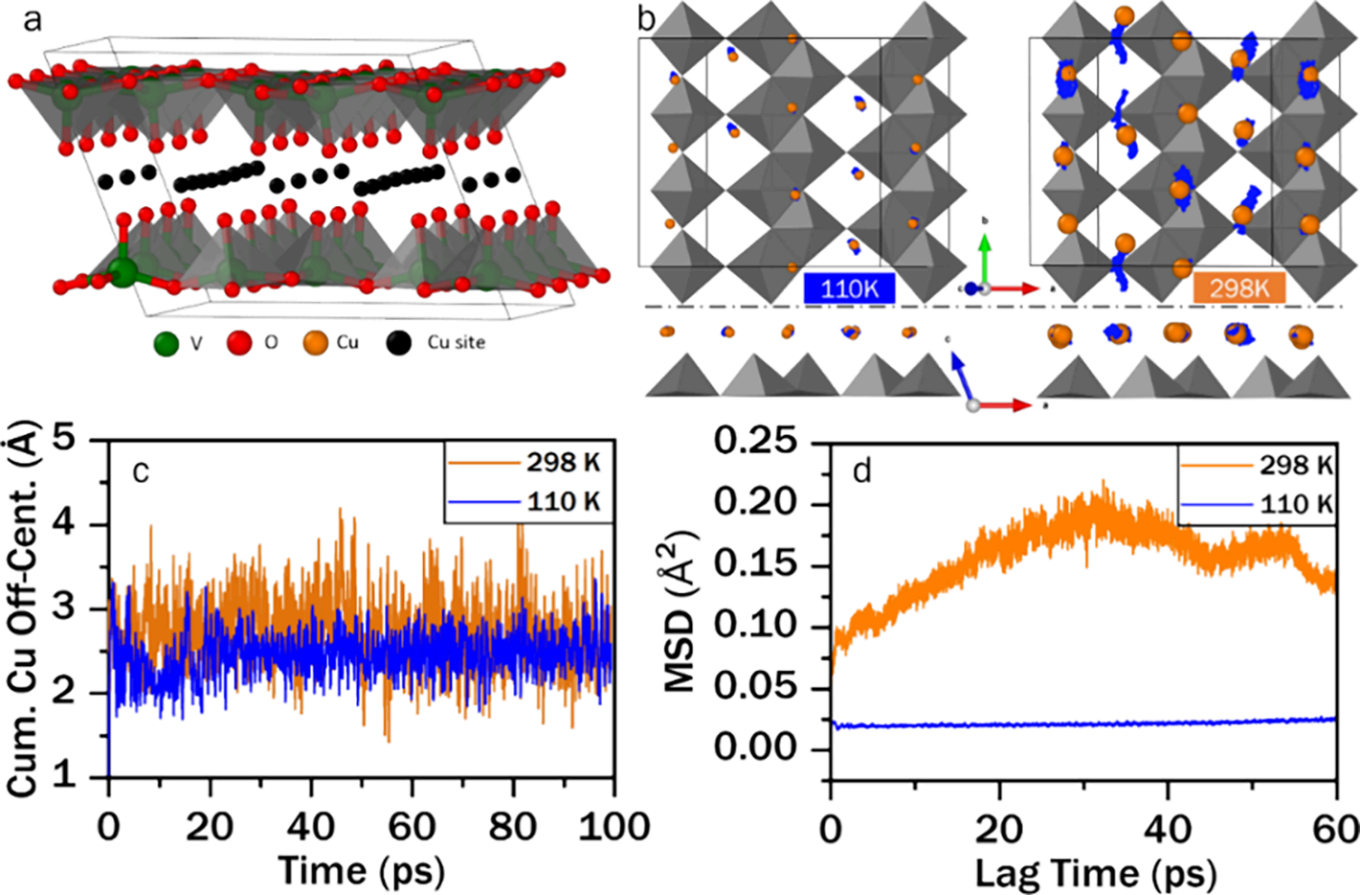
**Evolution of atomistic structure of ε-Cu**_**0.9**_**V**_**2**_**O**_**5**_**as a function of Cu-ion off-centering.***(***a***) Simulation cell showing available copper sites used to determine Cu-ion starting positions, as determined from single-crystal diffraction. (***b***) Simulation cells at 110 K (left) and 298 K (right), viewed perpendicular to the ab plane (top) and along the b-axis (bottom), with Cu ions (orange) demarcated in their starting positions and trajectories marked in blue. (***c***) Time evolution of cumulative Cu-ion off-centering, indicating significantly higher local disorder at higher temperature. (***d***) Cu-ion mean squared displacement (MSD) vs lag time, suggesting a drastic difference in long-range Cu-ion mobility between simulation temperatures*.

To understand how Cu-ion mobility underpins the observed conductance switching, ab initio molecular dynamics (AIMD) simulations have been performed to map possible ion trajectories around their average positions shown in Figure 4a. It is immediately apparent from the Cu-ion migration trajectories (blue traces) shown in Figure 4b for AIMD simulations run at 110 K (left) and 298 K (right) that copper ions migrate readily between adjacent Cu2 sites along *b* at high temperature, and that this migration is suppressed in the insulating low-temperature state. The sinusoidal trajectory shape exhibited in the left Cu2 chain (hosting a Cu-ion vacancy induced by having a substoichiometric amount of copper) is in remarkable agreement with the orientation of the thermal ellipsoid orientation shown in Figure 3b, and further corroborates the assignment of intersite Fourier density in Figure S6 to itinerant copper ions. Indeed, this also accounts for the thermal displacement parameters discussed above. The cumulative copper-ion off-centering, equal to the sum of all copper-ion displacements from the nearest crystallographic site (as depicted in Figure S8c), is plotted versus time in Figure 4c, whereas the copper-ion mean squared displacement is plotted versus lag time in Figure 4d.

The insulating low-temperature state exhibits much lower cumulative off-centering and mean squared displacement over the time scale of the simulation, which is consistent with the trajectories shown in Figure 4b. The AIMD results thus corroborate the Cu-ion short-range order/disorder as also observed in single-crystal diffraction as the fundamental origin of conductance switching.

### Electronic Structure Basis for Conductance Switching

**Figure 5:**
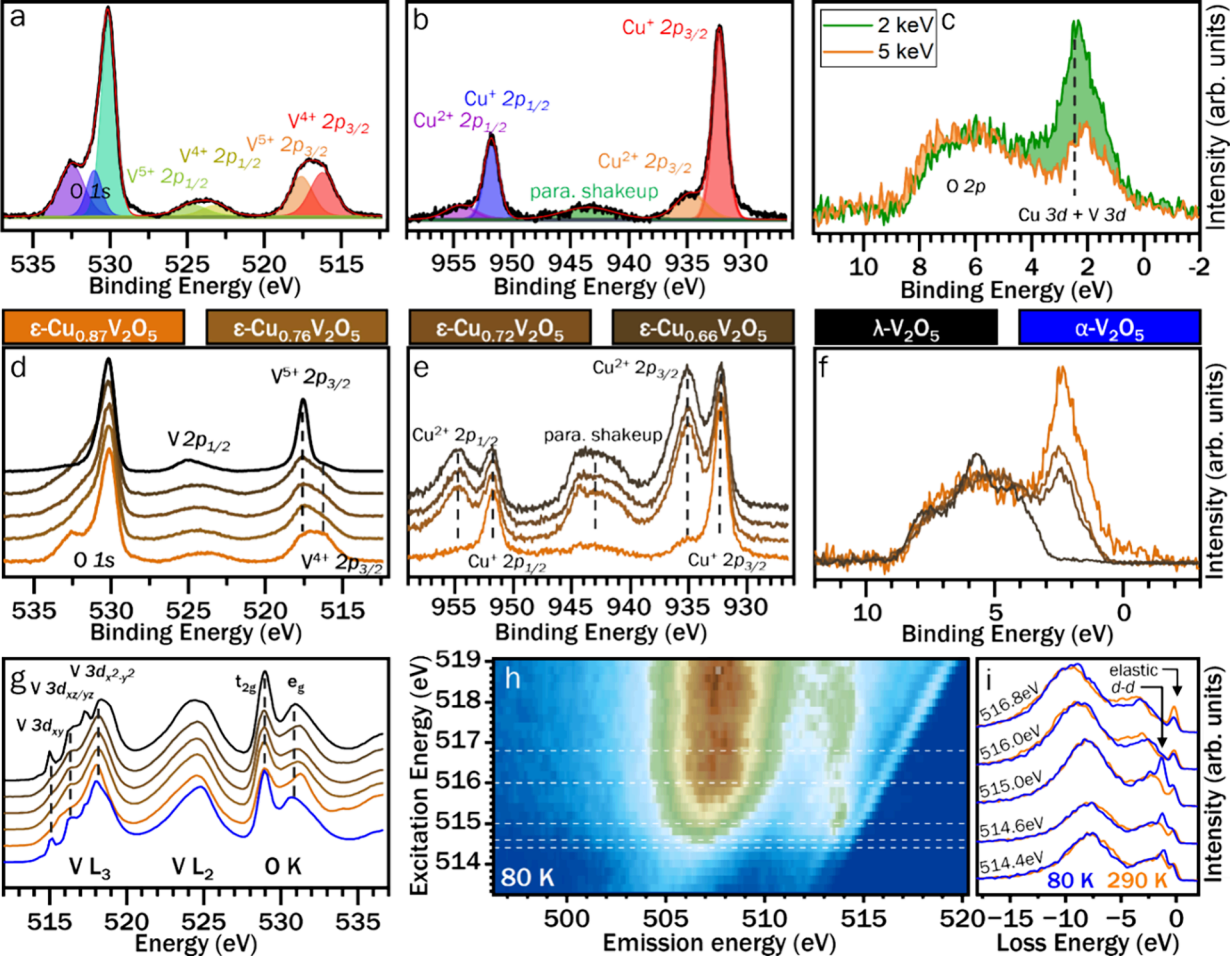
**Electronic Structure Characterization by X-ray Spectroscopies.** (**a-c***) HAXPES spectra collected at 2 keV excitation energy (unless indicated otherwise) for* ε*-Cu*_*0.9*_*V*_*2*_*O*_*5*_*at V 2p, O 1s (***a***) and Cu 2p (***b***) core levels, and at the valence band (***c***). (***d-f***) HAXPES spectra of partially deintercalated* ε*-Cu*_*x*_*V*_*2*_*O*_*5*_*samples and fully deintercalated* λ*-V*_*2*_*O*_*5*_*, collected at 2 keV excitation energy, at V 2p, O 1s (***d***), and Cu 2p (***e***) core levels, and at the valence band (***f***). In addition to the expected Cu*^*+*^*peak at 931.9 eV, the Cu 2p core level spectrum of* ε*-Cu*_*0.9*_*V*_*2*_*O*_*5*_*(*Figure 5***b****) exhibits a small peak at 934.6 eV, consistent with Cu*^*2+*^*as also observed in other copper vanadium bronzes.*^58^*The broad feature centered ca. 943 eV is assigned to paramagnetic shakeup processes and provides additional evidence of d*^*9*^*-configuration Cu*^*2+*^*.*^59^*(***g***) V L*_*2*_*-, L*_*3*_*- and O K-edge XANES spectra of* α*-V*_*2*_*O*_*5*_*, as-prepared* ε*-Cu*_*0.9*_*V*_*2*_*O*_*5*_*, partially deintercalated* ε*-Cu*_*x*_*V*_*2*_*O*_*5*_*, and completely deintercalated* λ*-V*_*2*_*O*_*5*_*. The narrowing of the e*_g_* O K-edge feature observed for ε*-Cu*_*0.9*_*V*_*2*_*O*_*5*_*as compared to* λ*-V*_*2*_*O*_*5*_*indicates a decrease of vanadium off-centering with increasing vanadium reduction.*

We next use Hard X-ray Photoelectron Spectroscopy (HAXPES) to characterize the charge states and valence band electronic structure of ε-Cu_0.9_V_2_O_5_. In addition, in order to disentangle the contributions of copper- and vanadium-centered states to the electronic structure of ε-Cu_0.9_V_2_O_5_, we performed both partial and complete copper deintercalation reactions, as detailed in the Experimental Section.^46^ Rietveld refinement of powder X-ray diffraction data and Energy-Dispersive X-ray Spectroscopy (EDS) have been used for structural and compositional characterization of the deintercalated products (Supporting Figures S9 and S10, Supporting Tables S6 and S7) and confirm near-complete copper removal for λ-V_2_O_5_ near limits of detection. Figure 5a shows the O 1*s* and V 2*p* core level spectra measured for ε-Cu_0.87_V_2_O_5_. As expected from the Cu stoichiometry, the V 2*p*_*3/2*_ core level feature exhibits roughly equal V^4+^ (at 515.9 eV) and V^5+^ (at 517.3 eV) contributions.^47,48^ The O 1*s* feature exhibits peaks centered at 530.0 and 530.9 eV, attributed to bridging and vanadyl oxygens, respectively,^49,50^ as well as a peak at 532.4 eV. In the O 1*s* spectra of the deintercalated samples (Figure 5d), this high binding energy feature is assigned to Cu–O interactions, which is further supported by the near absence of this shoulder in fully deintercalated λ-V_2_O_5_. The Cu 2*p* core-level HAXPES spectrum shows Cu below detectable limits corroborating near-complete decupration of λ-V_2_O_5_. Contrary to a simple electron counting formalism, according to which V^5+^ ions are reduced to V^4+^ in proportion to Cu^+^ intercalation, the apparent V^4+^/ V^5+^ signal varies little with removal of Cu^+^ across the partially deintercalated samples. The concomitant increase in Cu^2+^ signatures in Figure 5e suggests that V oxidation states are “protected” by compensatory oxidation of remaining Cu^+^ ions to Cu^2+^, which in turn implies energetic overlap of Cu and V 3*d* orbitals near the Fermi level.

A significant advantage of synchrotron-based HAXPES over conventional laboratory XPS is the continuous tunability of the incident X-ray energy. The large differences in rates of photoemission cross-section decay with increasing X-ray energy between orbitals with different angular momentum (see Supporting Figure S9 can be leveraged to determine the orbital character of overlapping features in the valence band.^51−53^Figure 5c contrasts valence band spectra of ε-Cu_0.9_V_2_O_5_ acquired at incident energies of 2 and 5 keV. At higher incident energy, the diminished intensity of the sharp feature at BE = 2 eV indicates that it comprises predominant contributions from high-angular-momentum orbitals, i.e. Cu 3*d* states. In addition, partially filled V 3*d*_*xy*_ orbitals have been shown to lie in close proximity to the Fermi level.^22,54^ Further support for the Cu 3*d*/V 3*d* assignments comes from the diminution and almost complete elimination of the 2 eV valence band feature for partially and completely deintercalated samples, respectively, shown in Figure 5f. The close energetic positioning of Cu 3*d*/V 3*d* near the Fermi level is the distinctive origin of the role of Cu-ions in mediating metal—insulator transitions in these systems. The valence band of λ-V_2_O_5_ has primarily O 2*p* character (hybridized with V 3*d*) but shows no intensity above ≈3 eV binding energy.

X-ray Absorption Near-Edge Spectroscopy (XANES) is a powerful element- and orbital-specific probe of the unoccupied density of states in a material, and thus serves as a complementary probe to HAXPES for examination of electronic structure. Figure 5g shows XANES spectra of as-prepared ε-Cu_0.9_V_2_O_5_ and deintercalated ε-Cu_*x*_V_2_O_5_ samples plotted alongside an α-V_2_O_5_ reference. The vanadium L_2_ and L_3_ absorption edges result from electron transitions from spin–orbit-split vanadium 2*p* orbitals into empty 3*d* orbitals.^55^ The V L_3_-edge displays a rich fine structure arising from the crystal-field splitting of 3*d* states.^56^ The intense V 3*d*_*xy*_ pre-edge feature observed for empty λ-V_2_O_5_ and the relatively reduced intensity observed for partially deintercalated ε-Cu_*x*_V_2_O_5_ samples indicates that Cu-ion insertion and concomitant vanadium reduction involves filling the low-lying V 3d_*xy*_ orbitals (which are diminished in intensity because of Pauli blocking).^57^ The oxygen K-edge XANES spectrum involves transitions from O 1*s*-derived states into (i) O 2*p*_*x*_ and 2*p*_*y*_ states engaged in π interactions with V 3*d t*_*2g*_ states (centered at 529 eV), and (ii) O 2*p* states engaged in σ* interactions with V 3*d e*_*g*_ orbitals centered at (531 to 532) eV.^47^

The relative diminution of the *t*_2g_ feature from λ-V_2_O_5_ to partially intercalated ε-Cu_*x*_V_2_O_5_ to pristine ε-Cu_0.87_V_2_O_5_ indicates increased V 3*d*_*xy*_ band filling with increased copper content. The e_g_* feature is broadened by splitting of lower-energy V 3*d*_*x*_2_–*y*_2 and higher-energy 3*d*_*z*_2 contributions due to the off-centering of the vanadium center from ideal octahedral coordination and concomitant loss of local symmetry.^60^ V and Cu K-edge X-ray absorption spectra collected on as-prepared and deintercalated materials are shown in supporting Figure S12 and provide further corroboration for the energetic overlap of Cu and V 3*d* states.^61^ The k^3^-weighted Fourier transforms of the V and Cu K-edge EXAFS spectra of λ-V_2_O_5_ and ε-Cu_*x*_V_2_O_5_ samples and IFEFFIT structure model fits are shown in supporting Figure S12(c-f) and Tables S8 and S9. Based on HAXPES and XANES measurements, the states in closest proximity to the Fermi level have predominantly Cu 3*d* and filled V 3*d* character, whose filling and overlap mediate conductance switching upon Cu-ion rearrangement driven by temperature or voltage.

Resonant inelastic X-ray scattering (RIXS) measures X-ray emission and inelastic scattering excited at a series of excitation energies selected across an absorption edge.^62^ Comparison of the positions of RIXS features at different excitation energies allows fluorescence processes (at constant emission energy) to be differentiated from lower-energy excitations (at constant energy loss) such as intraband and ligand-to-metal electron transitions. Figures 5h and 5**i** respectively show the V L_3_-edge RIXS spectral map of ε-Cu_0.9_V_2_O_5_ measured at 80 K, and stacked RIXS cuts of ε-Cu_0.9_V_2_O_5_ measured at both 80 and 290 K for a detailed comparison. The spectrum is dominated by fluorescence features arising from the decay of V-hybridized O 2*sp* states in the valence band (L_α_ emission) and of V 3*d* orbitals into the V 2*p*_3/2_ core hole created by photoexcitation,^63^ and are consistent with the valence band HAXPES features at ca. 6 and 1 eV, respectively, in Figures 5c and 5**f**. Dashed horizontal lines indicate excitation energies for the stacked emission spectra in Figure 5i. Plotting on an energy loss scale reveals an inelastic feature at 1.1 eV energy loss in the low-temperature spectra excited near the V 3*d*_*xy*_ resonance, attributed to on-site V 3*d*-V 3*d* electron transitions. The disappearance of this feature at ambient temperature is consistent with the delocalization of V 3*d*_*xy*_ electrons in the conductive state.^22^

**Figure 6:**
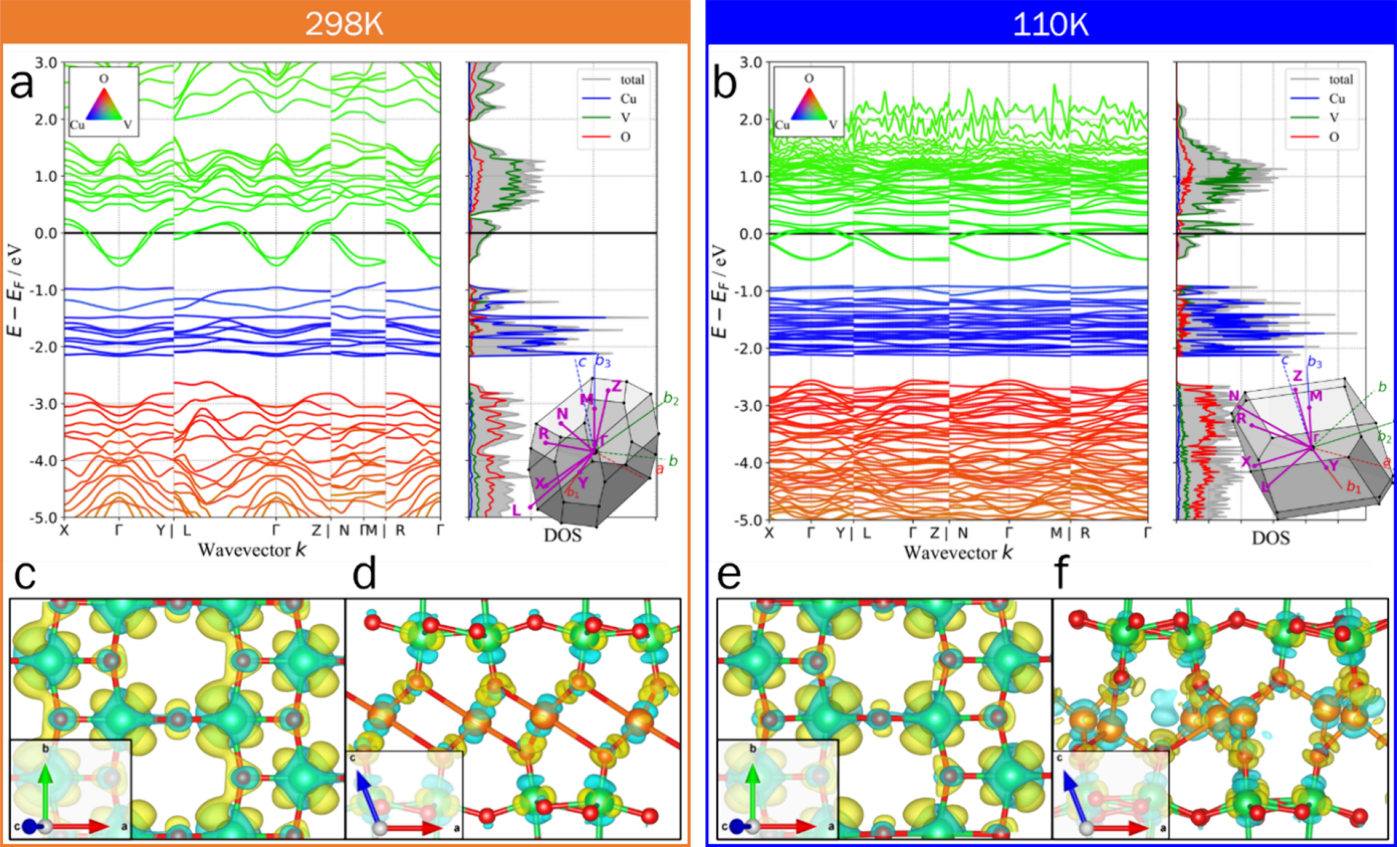
**Ground-state atom-projected ε-Cu**_**0.9**_**V**_**2**_**O**_**5**_**band structure.** (**a, b***) Band structure of* ε*-Cu*_*0.9*_*V*_*2*_*O*_*5*_*calculated at 298 K (***a***) and 110 K (***b***). Brillouin zone maps (insets) labeled with simulation cell (b*_*i*_*) and monoclinic cell (a, b, c) basis vectors. Dispersion of the V 3d*_*xy*_*band spanning the Fermi level shows a marked decrease at low temperature, consistent with localization of conduction electrons on V-sites. Notably, at 110 K, dispersion increases along***M**(perpendicular to the layer-stacking direction c*) possibly due to increased interaction between Cu-ion and V_*2*_*O*_*5*_*sublattices. (***c-f***) Charge density difference isosurfaces at 298 K (***c, d***) and 110 K (***e, f***) with positive and negative values rendered in yellow and blue, respectively. (***c***) and (***e***), drawn at 0.002 eÅ*^*–3*^*, show that electron density donated from Cu to the V*_*2*_*O*_*5*_*sublattice resides in V 3d*_*xy*_*-like states distorted to overlap with neighboring O-centered states and form conductive chains along b. (***d***) and (***f***), drawn at 0.01 eÅ*^*–3*^*, suggest an antibonding character to V 3d – O*_*V*_interactions along c* contrasted with the bonding character of in-plane V – O interactions.

Detailed electron band structure calculations performed on ground-state atomic configurations of ε-Cu_0.9_V_2_O_5_ at ambient and cryogenic temperatures reveal additional aspects of electronic structure modulation. Vanadium-centered 3*d* bands near the Fermi level exhibit a dispersive parabolic shape at room temperature (Figure 6a) characteristic of delocalized electrons, particularly in *k* directions lying in the *ab* plane. The same states are much less dispersive at 110 K along the *ab* plane due to localization on vanadium sites (Figure 6b). In several other layered materials, such band flattening near the Fermi level strengthens interactions between localized electrons and drives the formation of correlated phases with drastically altered physical properties.^64,65^ Since the effective mass is inverse proportional to band curvature, the change in band curvature induced as a result of Cu-ion reordering is likely to substantially modify electron mobilities. While the high carrier concentrations have thus far precluded reliable Hall measurements, future work will use angle-resolved photoemission spectroscopy to directly probe temperature- and voltage-induced modulation of band curvature and to explore whether band flattening strongly suppresses carrier mobilities.

Charge density difference maps were prepared by subtracting the calculated charge densities of the isolated, charge-neutral Cu and V_2_O_5_ sublattices from the that of ε-Cu_0.9_V_2_O_5_, and thus represent the redistribution of electron density consequent to V_2_O_5_ reduction and Cu oxidation. Accumulation of electron density in V 3*d*_*xy*_ orbitals in the *ab* plane is clearly visible as yellow lobes around vanadium atoms in Figure 6c and 6**e**_._ Similar features around bridging oxygens corroborate the covalency of V – O bonds, and their orientation suggests that π-type V 3*d* – O 2*p* overlap underpins in-plane electron delocalization. Figure 6d and 6**f** show the expected charge transfer from Cu layers to V_2_O_5_ slabs. Polarization of the vertical vanadyl bonds results from lower V – O_V_ bond covalency at reduced vanadium centers. At the same time, distortion of charge density lobes around O_V_ toward coordinated Cu cations arises from strong Cu *3d* – O_V_ interactions. The interplay between V – O and Cu – O covalency across V – O_V_ – Cu units mediates the observed coupling between Cu-ion order and V *3d* electron delocalization.

## Conclusions

Considerable recent attention has focused on the discovery of low-entropy-dissipative mechanisms and materials for neuromorphic computing. However, in most cases, detailed understanding of atomistic mechanisms and their implications for electronic structure remains obscure. In this work, we unveil sharply abrupt discontinuous conductance switching representing a transition from an antiferromagnetic insulator to a paramagnetic metal in ε-Cu_0.9_V_2_O_5_. Distinctively, we’ve fashioned nonlinear dynamical oscillators from millimeter-sized single crystals and used single-crystal diffraction to observe structural modifications underpinning conductance switching at an atomistic scale. We observe superlattice ordering of Cu ions between [V_4_O_10_] layers at low temperatures, which induces charge and spin ordering along the vanadium oxide framework, stabilizing an insulating state. AIMD simulations and mapping of superlattice reflections evidence the thermally activated mobility of Cu ions. Increasing temperature melts the superlattice order and yields charge delocalization. X-ray absorption and emission spectroscopies, assigned with the aid of electronic structure calculations and measurements of partially and completely decuprated samples, demonstrate the profound electronic structure implications of Cu-ion mobility. The narrowing of bandwidths mediated primarily by overlap of V 3*d*_*xy*_ states near the Fermi level drives the transformation to an insulating state. The results demonstrate a clear link between neuromorphic function and subtle structural distortions and resulting large modulations of electronic structure. Atomistic understanding of mechanisms of conductance switching paves the way to the rational design of active elements such as through cointercalation and site-selective modification. Future work will explore direct thermal probes of the transition including heat capacity measurements and expand systematic understanding of how oscillator performance is modified by dimensional reduction. Scaling these layered materials to ultrathin dimensions further holds opportunities for exploration of truly single-domain phenomena and for examining the influence of domain pinning.

## Experimental Section

### Preparation of ε-Cu_*x*_V_2_O_5_ Powders and topochemical Decupration

3.0435 g V_2_O_5_ powder (Sigma-Aldrich ≥99.6%) and 0.9567 g Cu metal powder (Sigma-Aldrich 99%) (approximately 1:0.9 mol ratio) were ball-milled together and loaded into a silica ampule, which was evacuated to 1.3 × 10^–1^ Pa and sealed using an acetylene torch. This vessel was heated to 823 K for 72 h in a muffle furnace to react. After cooling, the resulting black powder was ground in a mortar and pestle, sealed, and heated to 823 K for 72 h a second time to anneal. Topochemical decupration was achieved by the slow addition of a nominal 0.3 mol/L NO_2_BF_4_ (Sigma-Aldrich ≥95%) solution in anhydrous acetonitrile to a stirred acetonitrile suspension of ε-Cu_0.9_V_2_O_5_ in an Erlenmeyer flask under argon atmosphere, in amounts appropriate to achieve the desired degree of Cu removal. Complete removal of copper (to stabilize the λ-V_2_O_5_ polymorph)^46^ required a significant excess of oxidizer (ca. 2.5 equiv), likely because at high degrees of decupration, oxidation of dissolved Cu^+^ species to Cu^2+^ competes with further copper removal from the solid, a conclusion supported by the blue-green color of the supernatant after full decupration.

### Preparation and Exfoliation of Single Crystals of ε-Cu_0.9_V_2_O_5_

ε-Cu_0.9_V_2_O_5_ powder was loaded into a silica ampule which was evacuated to 1.3 × 10^–1^ Pa and sealed using an acetylene torch. This vessel was heated at 973 K for 3 h in a muffle furnace to melt, then cooled to 823 K at a rate of 0.5 K/h, then cooled to ambient temperature under furnace momentum to produce long, lustrous black plate crystals (Figure 1e). Single-crystal diffraction (*vide infra*) determined that the largest face corresponds to the (001) crystalline direction, whereas the longest direction corresponds to (010).

For exfoliation, ε-Cu_0.9_V_2_O_5_ crystals were affixed to an SEM stub using carbon tape, with the largest face (corresponding to the 001 plane) aligned parallel to the tape surface. Crystals were exfoliated by pressing a second piece of carbon tape onto the top of the crystals using a spatula and pulling the tape off. This procedure was repeated six times with small lateral movements between applications to spread exfoliated sheets across the carbon tape. The exfoliated crystals were imaged using a TESCAN FERA-3 scanning electron microscope (SEM) at a 9.3 mm working distance with an accelerating voltage of 5 kV.

### Electrical Transport and Oscillator Measurements

For electrical transport and oscillator measurements, ε-Cu_0.9_V_2_O_5_ single crystals were adhered onto an electrically insulating glass substrate using an insulating adhesive (GE varnish) and mounted on a cryostat insert with four-contact (for resistance—temperature) or two-contact (for current—voltage) electrodes spanning the length of the crystal (measuring transport along the longest axis, corresponding to the crystallographic *b-*direction) using Ag conducting paste. The temperature was varied from 50 to 300 K in a closed-cycle Janis/Lakeshore cryostat controlled by a Lakeshore 336 temperature controller operating in PID mode and was varied at rates of (1.0 to 1.5) K/min. Voltage was supplied by a Keithley 2450 source, with current limited to several milliamps to minimize Joule heating and crystal damage. *R* (*T*) and *I* (*V*) profiles were measured using either a Keithley 2450 or two SR7265 digital lock-in amplifiers and were automated using LabVIEW and MeasureLink software, whereas oscillations were recorded using a GW Instek GDS-2062 oscilloscope.

### Electrothermal Oscillator Simulations

Capacitance-dependent electro-thermal oscillations were simulated using a physics-based compact model that combines the material’s electrical and thermal properties with the nonlinear dynamics described by local activity theory. The model quantitatively connects intrinsic material properties to key performance parameters, including frequency, amplitude of electrical and thermal oscillations, and power. The compact model, previously described in [2], uses a coupled system of two first-order differential equations; 1) Kirchhoff voltage law, 2) Joule heating and Newton’s law of cooling. Simulations were performed with Runge–Kutta numerical integrator. The model is based on the following assumptions; (i) that quantities are uniform throughout the device, (ii) negligible series resistor and (iii) simplified thermal transport. The active material’s temperature-dependent electrical conductivity was based on the values shown in Figure 1a. Temperature-dependent thermal conductivity and specific heat capacity were obtained from Wang et al.^66^ and Drake et al.,^67^ respectively, with each data set fitted to physics-based models, providing analytical representations of their behavior.

### X-ray Diffraction

Powder X-ray diffraction was performed in Bragg–Brentano geometry using a Bruker D8 Endeavor diffractometer (Cu Kα, λ = 1.5418 Å source, 40 kV voltage, 25 mA current) with a Lynxeye detector. Rietveld refinements of powder diffraction data were performed using GSAS-II structure analysis software.^68^

Single crystal X-ray diffraction data were collected using a Rigaku Synergy Ag diffractometer with X-rays generated by an Ag anode tube source, (Ag Kα, λ = 0.56087 Å), controlled by Rigaku ChrysAlisPro software. Sample temperature control was achieved using a cold N_2_ stream. Crystal faces were indexed according to the procedure implemented within ChrysAlisPro: a video of the crystal was collected during φ-rotation and crystal faces visible perpendicular to the image plane were selected and identified by comparison to the previously collected unit cell and orientation (UB) matrix (see Figure S13). Frame integration, data reduction and absorption correction were performed using ChrysAlisPro software. Structure solution and least-squares refinement were performed using ShelXT (by the Intrinsic Phasing method) and ShelXL software, respectively, both implemented within the Olex2 software environment. Structures were visualized using VESTA 3 software.^69^

### Hard X-ray Photoelectron Spectroscopy

HAXPES measurements were performed at National Institutes of Standards and Technology (NIST) beamline 7-ID-2 at the National Synchrotron Light Source II (NSLS-II) at Brookhaven National Laboratory. X-ray energies were selected using a Si (111) double-crystal monochromator. Core levels were collected at 2 keV excitation and valence bands at 2 and 5 keV excitation energies. The 2 and 5 keV spectra were collected with a hemispherical electron energy analyzer oriented perpendicular to the beam axis, with 200 and 500 eV pass energies, respectively; and a 50 meV step size. Beam spot position on samples and electron flood gun current and voltage were carefully controlled to mitigate sample charging.

### X-ray Absorption Near-Edge Structure Spectroscopy

XANES measurements were made at NIST beamline 7-ID-1 at NSLS-II. Incident beam energy was selected using a variable line spacing plane grating monochromator. An electron flood gun was used to prevent sample charging. Partial electron yield signals were collected using a channeltron electron multiplier under −300 V detector entrance grid bias and normalized to the incident beam intensity as measured by a freshly evaporated gold mesh. Spectra were preto-postedge normalized and energy-aligned to the V *3d*_*xy*_ pre-edge feature of a fresh α-V_2_O_5_ standard using the ATHENA software package.^70^

### Extended X-ray Absorption Fine Structure (EXAFS) Spectroscopy

V K-edge and Cu K-edge X-ray absorption spectroscopy (XAS) scans were acquired at beamline 7-BM at National Synchrotron Light Source II of Brookhaven National Laboratory. Samples were prepared by uniformly spreading powder onto a piece of polyimide (Kapton) tape. The polyimide tape was then loaded onto a sample holder, and 20 scans were performed at 30 s per scan and subsequently averaged to improve the signal-to-noise ratio. Before sample acquisition, the beamline was calibrated by placing metallic vanadium, copper, and lead foils, and measuring the edge position. Spectra were collected in both fluorescent and transmittance modes. The Athena program from the IFEFFIT package was used for data sanitization. Data in the k range of 2.5 Å^–1^ to 11.0 Å^–1^ was Fourier transformed to obtain R-space data. The R-space data was used to perform shell fitting. Fitting was performed for the major shells between R space = 1.1 Å to 4.0 Å. Multishell least-squares parameter fitting of V K-edge, and Cu K-edge EXAFS data was performed using the ARTEMIS module of the IFEFFIT software package.^70^ The photoelectron mean free path, scattering amplitude, and phase functions were calculated using the FEFF6 program. Atomic coordinates and lattice parameters obtained from crystallography data were used to build initial models for EXAFS fitting.

### Resonant Inelastic X-ray Scattering

RIXS spectra were collected at the Advanced Light Source beamline 8.0.1.1’s high-efficiency iRIXS endstation^71^ using linearly polarized (perpendicular to the scattering plane) radiation supplied by an undulator and spherical grating monochromator. Excitation energy was calibrated to the V L_3_ highest-intensity V L_3_ feature of an α-V_2_O_5_ standard, and emission energies were calibrated by applying a linear fit to the elastic (zero energy-loss) feature.

### First-Principles Calculations

The density functional theory (DFT) method implemented in the Vienna *ab initio* simulation package (VASP, version 5.4.4) was the calculation engine for all ab initio molecular dynamics simulations (AIMD) and electron structure calculations.^72−75^ Electronic correlation effects were modeled with the generalized gradient approximation proposed by Perdew, Burke, and Ernzerhof (GGA-PBE), using the projector augmented wave method (PAW) with a plane-wave basis expansion.^76,77^ Initial atomic positions for ε-Cu_0.9_V_2_O_5_ were from single-crystal structure solutions at 110 and 295 K. Band structures, density of states, and charge density difference calculations were performed with a cutoff energy of 500 eV and a Monkhorst–Pack k-point sampling grid of 5 × 5 × 5. The convergence criteria for electronic relaxation was set to <10^–5^ eV. For AIMD calculations the cutoff energy was increased to 520 eV.^76,78,79^ All geometry optimizations were performed with a break condition for the electronic self-consistent loop set to 10^–4^ eV, and using the conjugated gradient algorithm to update ionic positions (0.05 eV/Å).

### Magnetic Measurements

Magnetic measurements of ε-Cu_0.9_V_2_O_5_ powders were carried out on a Quantum Design Magnetic Property Measurement System using the Quantum Design superconducting quantum interference device (SQUID) magnetometer option. Both zero-field cooled (ZFC) and field-cooled (FC) measurements were performed in the temperature range of (2 to 400 K) with an applied field up to 0.1 T. Field-dependent magnetization measurements were performed at 2 K and above room temperature under an applied magnetic field ranging from −7 T to +7 T.

### Scanning Electron Microscopy and Energy-Dispersive Spectroscopy

Scanning electron microscopy images and energy-dispersive X-ray (EDX) spectra were collected at 20 kV accelerating voltage using a JEOL JSM - 7500F FE (RRID: SCR_022202) instrument equipped with an Oxford Instruments Ultim Max 40 silicon drift detector, from samples gently pressed onto carbon tape.

### Transmission Electron Microscopy

Transmission electron microscopy (TEM) imaging was performed on an image-corrected Thermo Fisher Scientific (TFS) Titan Environmental TEM operating at 300 kV. The beam current was limited to 1 nA to minimize beam damage. Scanning transmission electron microscopy (STEM) imaging was performed on a probe-corrected TFS Titan Themis operating at 300 kV with a 25 mrad convergence semiangle and beam current of 12 pA. High-angle annular dark-field (HAADF)-STEM images were acquired using inner and outer collection angles of 65 and 200 mrad, respectively. Powder samples were prepared by scraping ε-Cu_0.9_V_2_O_5_ particles directly onto carbon-coated TEM grids. TEM lamellae were prepared from single crystals of ε-Cu_0.9_V_2_O_5_ using a TFS Nova 600 Nanolab following standard focused ion beam (FIB) lift out procedures.

## Supporting Information Available

Checkcif report for ε-Cu_0.9_V_2_O_5_, CSD deposition #2373974; additional experimental results, including transport measurements, oscillator characterization and simulation, electron micrographs, EDS quantification, crystallographic details, and EXAFS spectral fitting results (PDF)
Diffractometer frame images showing the appearance of supercell reflections upon cooling and disappearance upon heating (Video S1) (MP4)
