# Supplementary material for: Atomistic Origins of Conductance Switching in an ε-Cu0.9V2O5 Neuromorphic Single Crystal Oscillator
Source: J Am Chem Soc. 2024 Dec 4;146(50):34536–50. doi: 10.1021/jacs.4c11968 (PMC11664580; doi:10.1021/jacs.4c11968)
Supplement: Supplementary file 1 — ja4c11968_si_001.pdf [file ja4c11968_si_001.pdf]

Supporting Information

Atomistic Origins of Conductance Switching in an ε-$Cu_{0.9}V_2O_5$ Neuromorphic Single Crystal Oscillator

John Ponis[1], Nicholas Jerla[2], George Agbeworvi[1], Saul Perez-Beltran[1,3], Nitin Kumar[2], Kenna Ashen[3], Jialu Li[4], Edrick Wang[4], Michelle A. Smeaton[5], Fatme Jardali[3], Sarbajeet Chakraborty[1], Patrick J. Shamberger[3], Katherine L. Jungjohann[5], Conan Weiland[6], Cherno Jaye[6], Lu Ma[7], Daniel Fischer[6], Jinghua Guo[4], G. Sambandamurthy[2]*, Xiaofeng Qian[3]*, Sarbajit Banerjee[1,3]*

1. Department of Chemistry, Texas A&M University, College Station, TX 77843, USA
2. Department of Physics, University at Buffalo, State University of New York, Buffalo, NY 14260, USA
3. Department of Material Science and Engineering, Texas A&M University, College Station, TX 77843, USA
4. Advanced Light Source, Lawrence Berkeley National Laboratory, Berkeley, CA 94720, USA
5. National Renewable Energy Laboratory, Golden, CO 80401, USA
6. Material Measurement Laboratory, National Institute of Standards and Technology, Gaithersburg, MD 20899, USA
7. National Synchrotron Light Source II, Brookhaven National Laboratory, Upton, NY 11973, USA

*sg82@buffalo.edu; jguo@lbl.gov; feng@exchange.tamu.edu ; banerjee@chem.tamu.edu

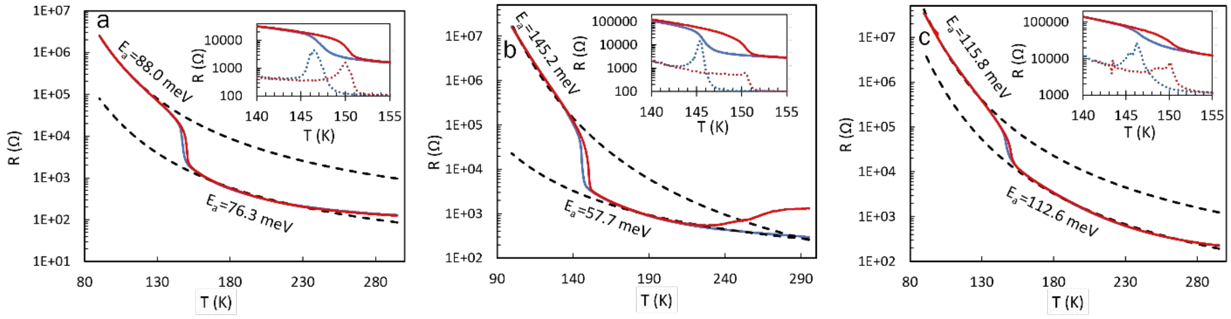

Figure S1. Transport Behavior in ε-$Cu_{0.9}V_2O_5$ Single Crystals.

(a-c) Electrical resistance *vs.* temperature for three different crystals. Pronounced conductance switching is observed in each case in the range of (145 K to 150 K). Note that the broadening of The increased resistance above 220 K shown in (b) is due to loss of contact because of crystal expansion and contraction during thermal cycling. Despite variations in activation energies derived from differences in density and carrier concentrations, the transition temperature and hysteresis width are remarkably consistent. The widths of the differential curves and differences in hysteresis derive from nucleation restrictions to initiation of insulating and metallic domains, which tend to be asymmetric for such materials and reflect in large measure the pinning of domains.

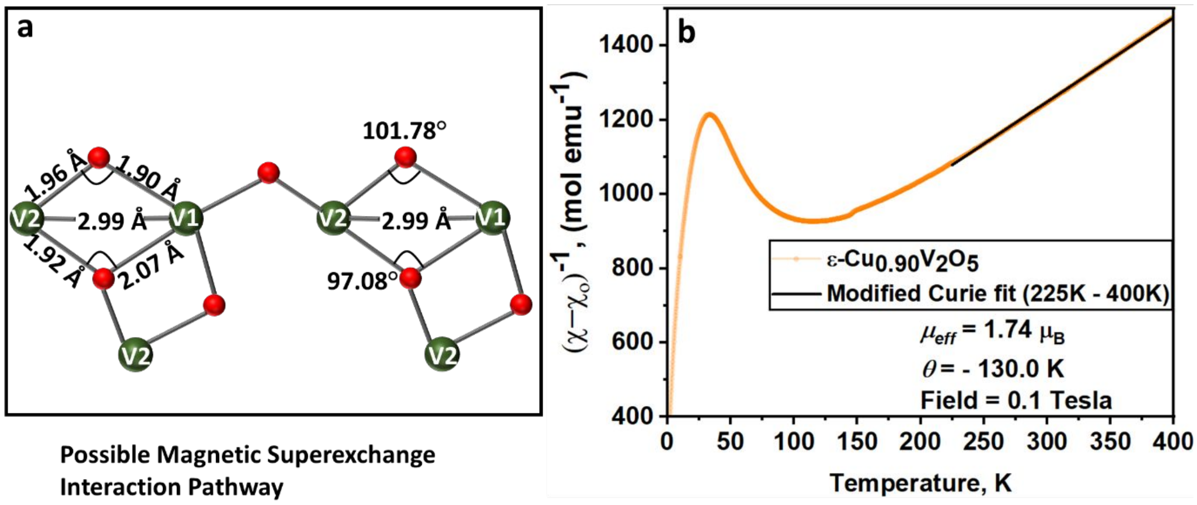

Figure S2. Magnetic Characterization of ε-$Cu_{0.9}V_2O_5$. (a) Possible magnetic superexchange interaction pathways in ε-$Cu_{0.9}V_2O_5$. (b) The inverse magnetic susceptibility has been fit to the modified Curie−Weiss law from 225 K to 400 K; the relevant fit parameters obtained are presented within the panel. The negative Weiss constant indicates AFM interactions derived from V(1)-O(2)-V(2) superexchange interactions. The extracted effective magnetic moment, μ_{eff} = 1.74 μ_B is consistent with the theoretical spin-only value for V^{4+} (1.73 μ_B, S = 1/2) reflecting partial reduction of vanadium. The upturn of susceptibility observed at ≈25K is attributed to ferromagnetic (FM) exchange coupling that causes V^{n+} ions across the [V_4O_{10}] slabs to exhibit a canted-spin arrangement.[1]

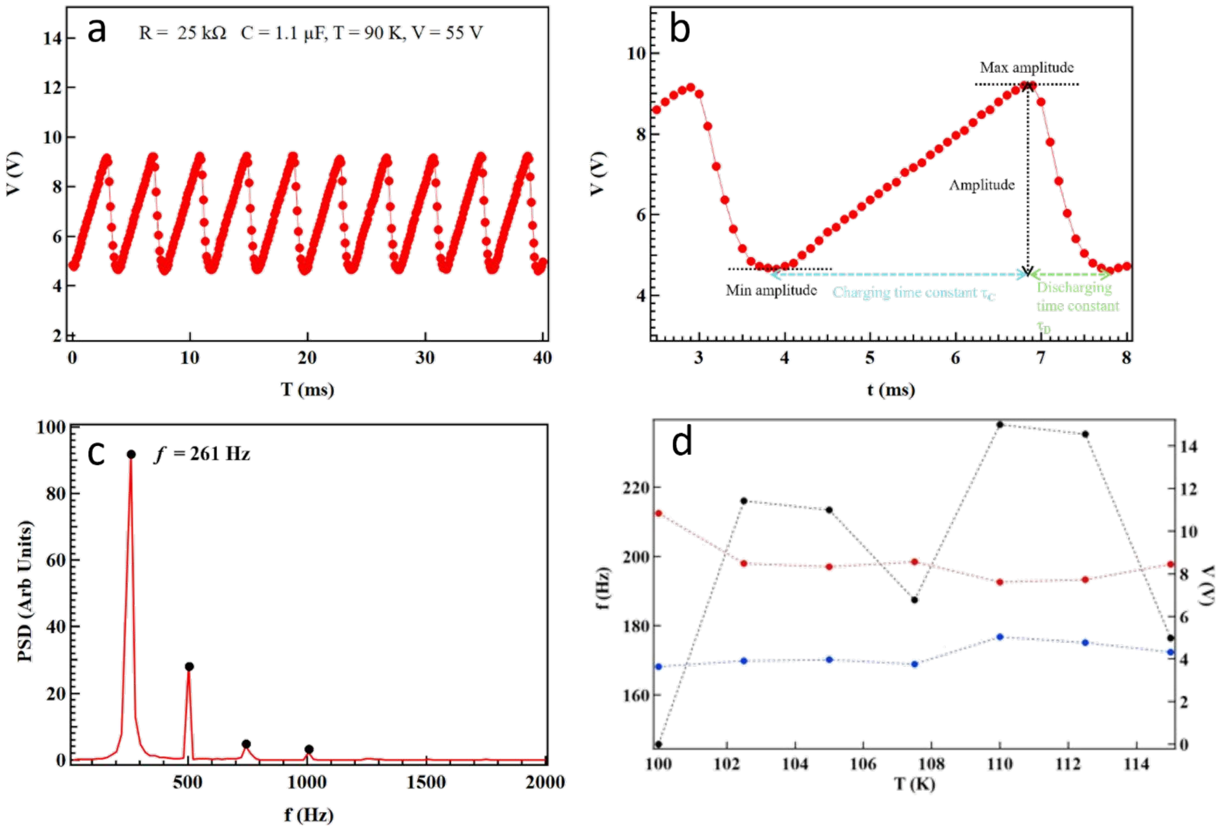

Figure S3: Additional Oscillator Characterization. (**a**) Oscillations exhibited by an example crystal under the specified conditions. (**b**) Diagrammatic depiction of amplitude and charging and discharging time constants for the oscillations shown in (a). (**c**) Fast Fourier transform (FFT) of (a), showing the fundamental oscillation frequency at 261 Hz and three harmonics marked with black dots. (**d**) Oscillation amplitude (maximum in red, minimum in blue) and frequency (black) versus sink temperature T_0. For a given set of circuit parameters, oscillations in ε-$Cu_{0.9}V_2O_5$ are stable only within a narrow temperature (and voltage) range, which is consistent with the first-order highly non-linear dependence of C_e, R_e, and R_t on T_0, across the temperature-induced structure and conductivity transition at (145 K to 150 K).

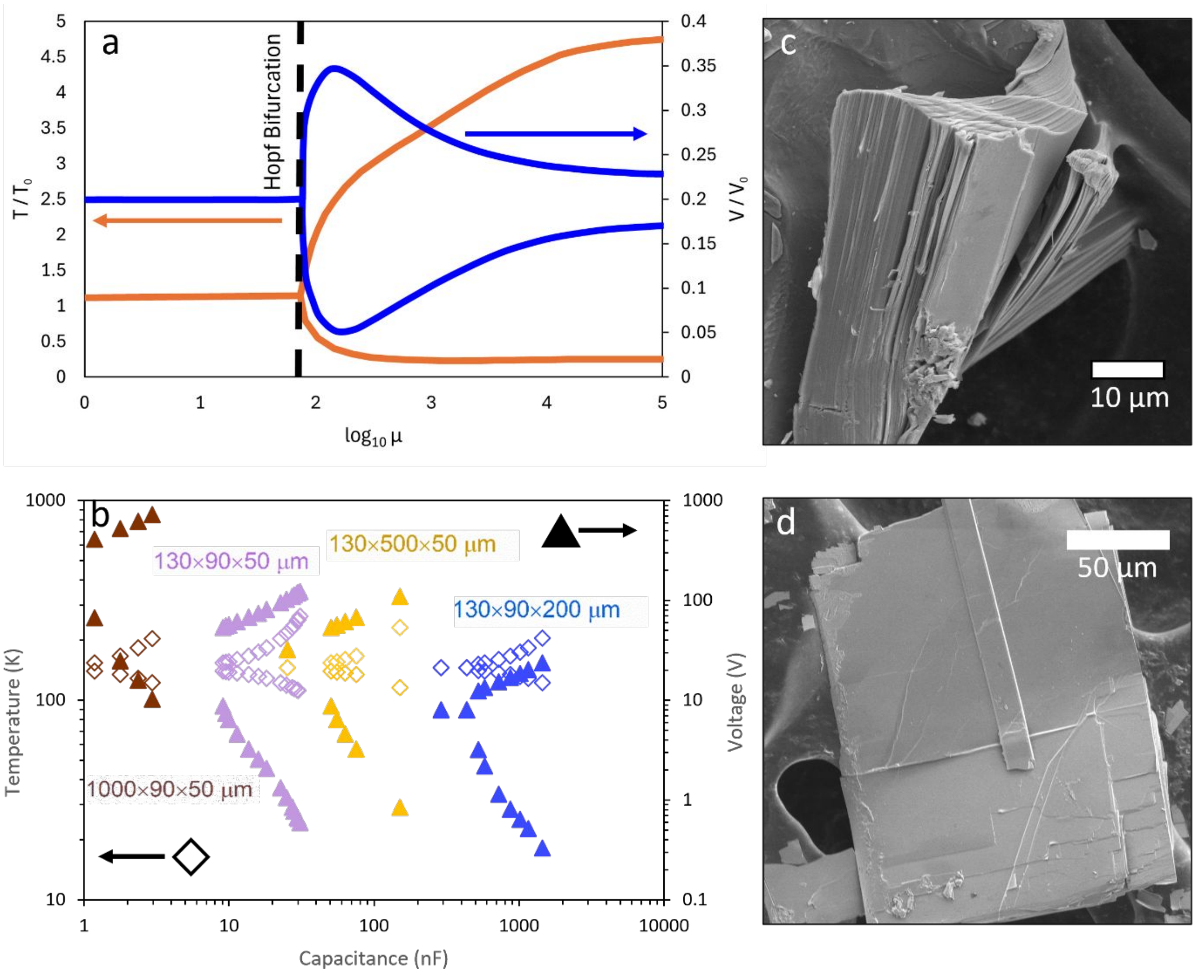

Figure S4: Electro-thermal Oscillator Theory, Simulation, and Geometry Modification (**a**) Predicted maximum and minimum voltage (blue) and temperature (orange) fluctuations versus μ for electro-thermal oscillators, adapted from Brown *et al*.[2] (**b**) Simulated values plotted vs. capacitance for several crystal geometries. (**c**) Side view of incompletely mechanically exfoliated crystal showing layer separation. (d) Tape-exfoliated single crystal with several smaller exfoliated sheets on surface and in periphery.

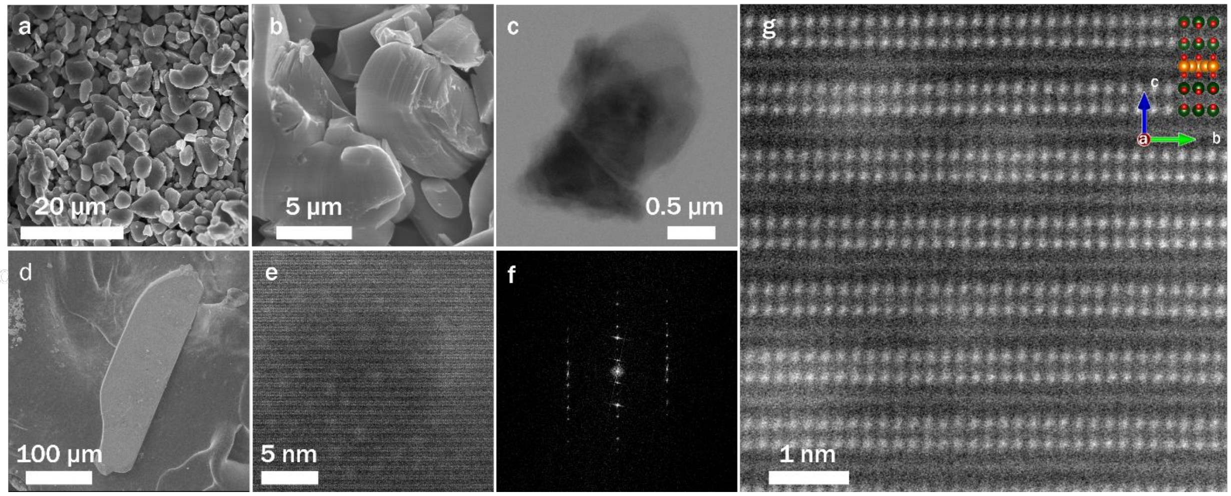

Figure S5. Electron microscopy characterization of ε-$Cu_{0.9}V_2O_5$

(a,b) SEM images of ε-$Cu_{0.9}V_2O_5$ powder. Striations on particle surfaces in (b) likely result from mechanically induced layer shear deformations. (c) TEM micrograph of a powder particle displaying multiple crystalline domains and visible layering. (d) SEM image of an ε-$Cu_{0.9}V_2O_5$ crystal. The visible surface is the (001) face and the longest direction is (010). (e,f) STEM image and Fourier transform, respectively, of the (100) projection of a lamella cut from a single crystal using a focused ion beam. The layered structure if the crystal is evident by the atomic lattice visible in (e) and by the wide horizontal (along b*) and narrow vertical (along c*) spacing of spots in (f). (g) STEM image of a similarly prepared lamella. Individual V atoms are visible and exhibit the double-layer motif indicated by the atomic structure overlay. Chains of Cu atoms along *b* are visible as horizontal stripes between [V_4O_{10}] layers.

Table S1. Crystal data and structure refinement for ε-$Cu_{0.9}V_2O_5$ at 293K, 20u5_1c_x2_f1_rt_auto, CSD deposition #2373974

**Identification code**	20u5_1c_x2_f1_rt_auto	
**Empirical formula**	Cu0.907 O5 V2	
**Formula weight**	239.07 g/mol	
**Temperature**	293 K	
**Wavelength**	0.56087Å	
**Crystal system**	Monoclinic	
**Space group**	*C 2/m*	
**Unit cell dimensions**	*a* = 11.7622(2)Å	α = 90°.
	b = 3.69080(10)Å	β = 111.568(2)°
	c = 8.9663(2)Å	γ = 90°.
**Volume**	361.990(15) $Å^3$	
Z	4	
**Density (calculated)**	4.395 g/mL	
**Absorption coefficient**	5.265 mm^{-1}	
**F(000)**	449	
**Crystal size**	0.77 × 0.23 × 0.09 mm^3	
**Theta range for data collection**	2.862 to 30.526°	
**Index ranges**	-19<=h<=19, -5<=k<=6, -15<=l<=15	
**Reflections collected**	31363	
**Independent reflections**	1126 [R(int) = 0.0563]	
**Completeness to theta = 19.665°**	0.997	
**Absorption correction**	Empirical using spherical harmonic crystal model	
**Max. and min. transmission**	1 and 0.616	
**Refinement method**	Full-matrix least-squares on F^2	
**Data / restraints / parameters**	1126 / 0 / 55	
**Goodness-of-fit on F2**	1.064	
**Final R indices [I>2sigma(I)]**	R1 = 0.0356, wR2 = 0.0930	
**R indices (all data)**	R1 = 0.0389, wR2 = 0.0967	
**Extinction coefficient**	-	
**Largest diff. peak and hole**	2.453 and -1.303 $eÅ^{-3}$	

Table S2. Atomic coordinates, occupancies, and equivalent isotropic displacement parameters ($Å^2$) for ε-$Cu_{0.9}V_2O_5$ at 293 K, 20u5_1c_x2_f1_rt_auto, CSD deposition #2373974. U_{eq} is defined as one third of the trace of the orthogonalized **U** tensor.

Atom	M	*x*	*y*	*z*	Occupancy	U_{eq} ($Å^2$)
V1	4	0.48229(3)	0.5	0.16862(4)	1	0.00987(11)
V2	4	0.78017(3)	0.5	0.16403(4)	1	0.00990(11)
O1	4	0.54447(19)	0.5	0.3668(2)	1	0.0196(3)
O2	4	0.82195(18)	0.5	0.3654(2)	1	0.0182(3)
O3	4	0.44008(15)	0	0.1422(2)	1	0.0126(3)
O4	4	0.79504(15)	0	0.1136(2)	1	0.0123(3)
O5	4	0.60887(15)	0.5	0.1101(2)	1	0.0126(3)
Cu1	2	1	0.5	0.5	0.969(3)	0.02758(18)
Cu2	4	0.75	0.75	0.5	0.423(2)	0.0334(3)

Table S3: Anisotropic displacement parameters ($Å^2$) for ε-$Cu_{0.9}V_2O_5$ at 293 K, 20u5_1c_x2_f1_rt_auto, CSD deposition #2373974. The anisotropic displacement factor exponent takes the form: $-2\pi^2 [h^2 a^{*2}U_{11} + ... + 2 h k a^* b^* U_{12}]$.

Atom	U_{11}	U_{22}	U_{33}	U_{12}	U_{13}	U_{23}
V1	0.00588(16)	0.01032(17)	0.01486(18)	0	0.00554(12)	0
V2	0.00673(16)	0.00966(17)	0.01501(18)	0	0.00599(12)	0
O1	0.0169(8)	0.0247(8)	0.0173(8)	0	0.0065(6)	0
O2	0.0124(7)	0.0253(8)	0.0183(8)	0	0.0072(6)	0
O3	0.0076(6)	0.0113(6)	0.0214(7)	0	0.0082(5)	0
O4	0.0086(6)	0.0104(6)	0.0192(7)	0	0.0069(5)	0
O5	0.0074(6)	0.0136(6)	0.0196(7)	0	0.0083(5)	0
Cu1	0.0125(2)	0.0480(4)	0.0198(3)	0	0.00315(17)	0
Cu2	0.0348(5)	0.0472(6)	0.0242(4)	0.0193(4)	0.0178(4)	0.0006(4)

Table S4. Select interatomic distances and bond angles grouped by central atom in ε-$Cu_{0.9}V_2O_5$ at 293 K, 20u5_1c_x2_f1_rt_auto, CSD deposition #2373974. Cis and trans subscripts denote position of space-group-equivalent oxygen atoms relative to vanadyl oxygens O_V.

			1-2-3 Angle (°), Atom 3			
**1-2 Distance (Å)**	**Atom 1**	**Atom 2**	**$O5_{cis}$**	**O3**	**O4**	**$O5_{trans}$**
1.654(2)	**$O1_V$**	**V1**	103.47(10)	97.24(6)	105.50(9)	178.93(9)
1.7509(16)	**$O5_{cis}$**	**V1**		100.03(5)		77.60(8)
1.9031(4)	**O3**	**V1**		151.72(10)	76.39(5)	82.54(6)
2.0725(17)	**O4**	**V1**				73.43(7)
2.3294(18)	**$O5_{trans}$**	**V1**			151.03(8)	
			**V2**			
2.9979(4)	**V2**	**V1**	75.985(11)			
			**O5**	**$O4_{cis}$**	**O3**	**$O4_{trans}$**
1.688(2)	**$O2_V$**	**V2**	97.89(9)	103.63(5)	101.19(9)	174.89(8)
1.8914(16)	**O5**	**V2**		96.91(5)	160.92(8)	77.00(7)
1.9233(5)	**$O4_{cis}$**	**V2**		147.28(10)	83.91(7)	77.23(5)
1.9616(17)	**O3**	**V2**				
2.3151(18)	**$O4_{trans}$**	**V2**			78.64(5)	
			**O2**	**O1**		
1.996(2)	**O2**	**Cu1**	180	85.84(6)	94.16(6)	
2.3591(13)	**O1**	**Cu1**		77.06(8)	102.94(8)	180
			**O2**	**O1**		
1.9428(18)	**O2**	**Cu2**	180	84.53(7)	95.47(7)	
2.4494(19)	**O1**	**Cu2**		180		
1.84540(5)	**Cu2**	**Cu2**				
			**Cu2**		**Cu1**	
	**V1**	**O1**	119.42(10)		116.81(6)	
	**V2**	**O2**	112.56(4)	129.97(9)	118.35(11)	
			**V1**	**V2**		
	**V1**	**O3**	151.72(10)	101.73(5)		
	**V1**	**O4**		97.16(5)		
	**V2**	**O4**		147.28(10)		

a

b

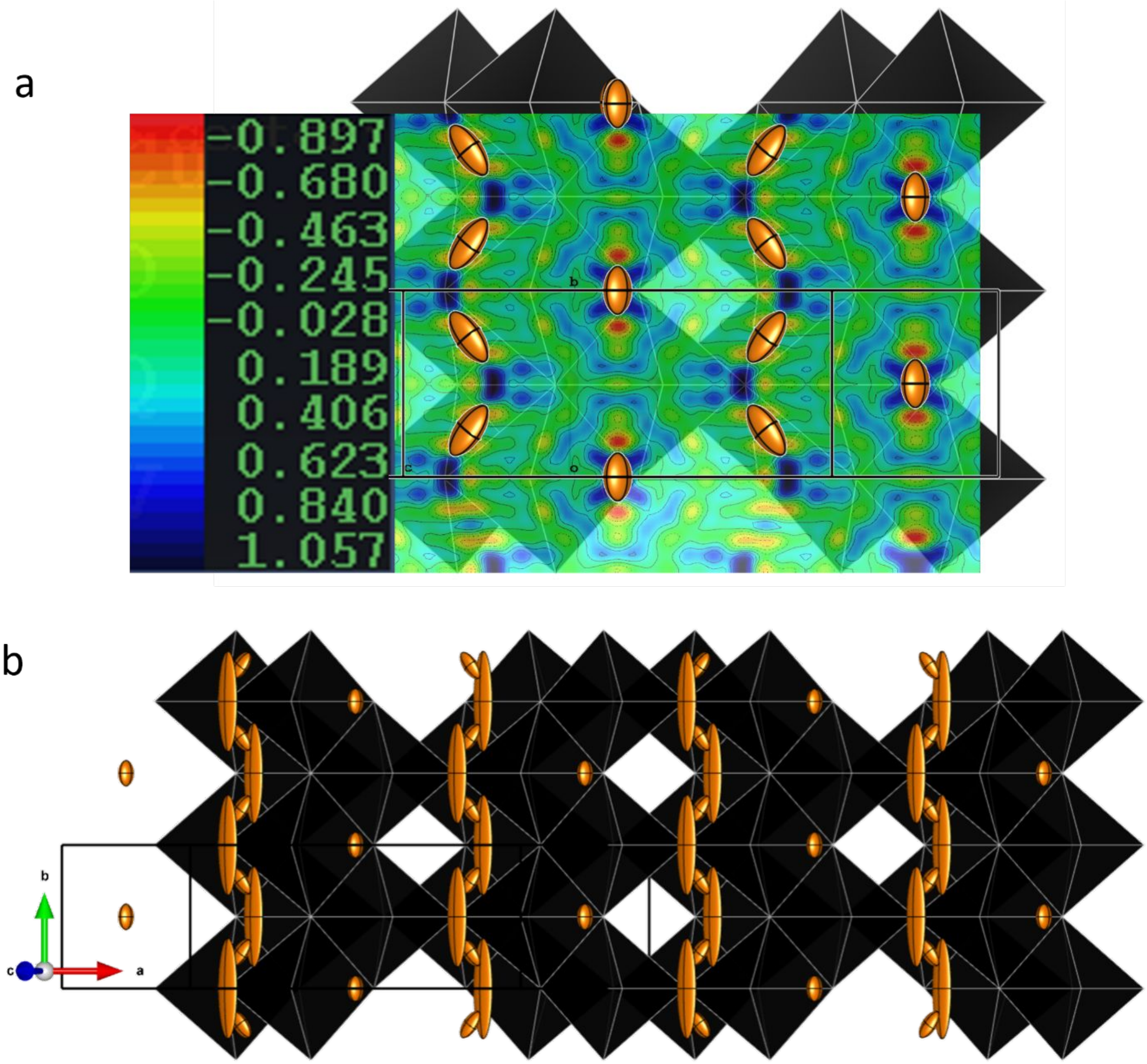

Figure S6. Crystallographic Evidence of Inter-site Cu-ion Migration

(a) Ambient-temperature structure of ε-$Cu_{0.9}V_2O_5$ with overlaid Fourier difference density map in the Cu-ion-containing plane. Blue regions indicate diffuse electron density ascribed to a high population of transient copper ions. (b) Structure with Cu-ions fit to inter-site electron density. The length of the resulting thermal ellipsoids indicates diffuse copper distribution along the *b* direction.

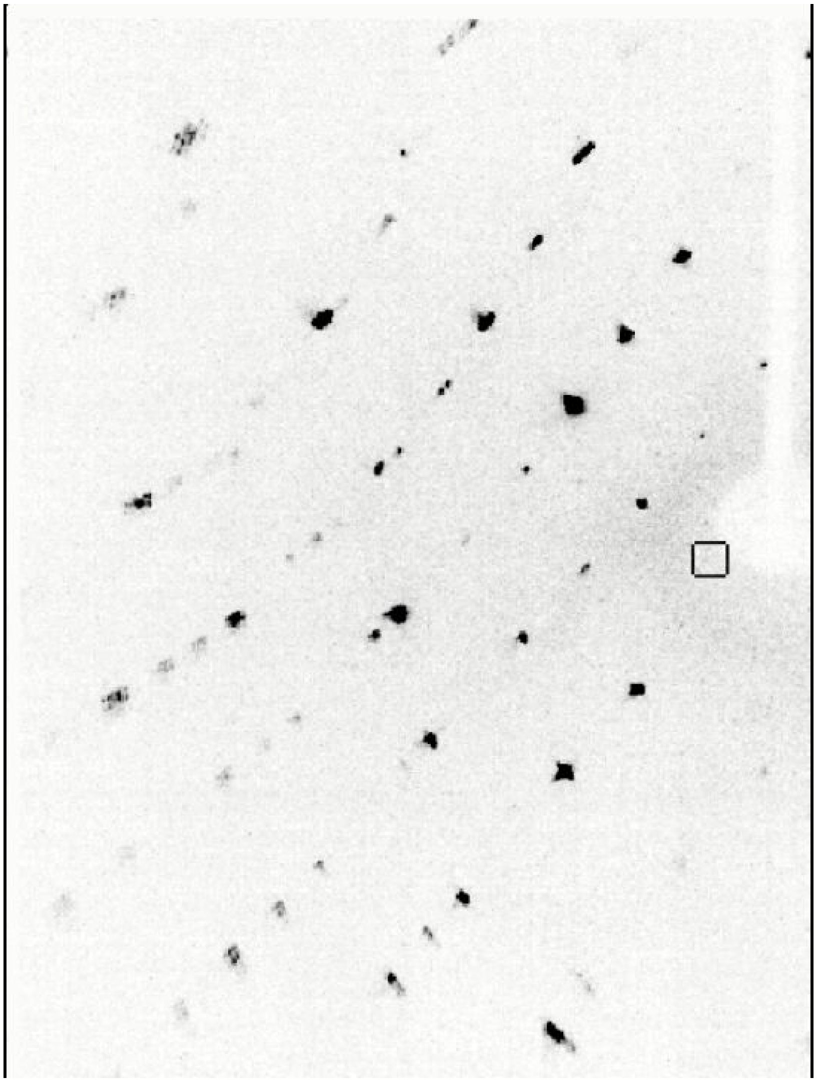

Video S1. Supercell Spot Appearance upon Cooling and Disappearance upon Heating

Cooling from 160 K to 115 K (at 1 K/frame to 140 K, then 5 K/frame to 115 K) induces the formation of a supercell with the appearance of accompanying supercell reflections. Subsequent heating to 160 K (at 5 K/frame to 145 K, then 1 K/frame to 160 K) eliminates the supercell and reflections, demonstrating the reversibility of the crystallographic transition.

Table S5. Bond Valence Sum calculations for Cu and V atoms in ε-$Cu_{0.9}V_2O_5$ at 293 K (left column) and 110K (center and right columns). Bond valences calculated according to the equation BV = $\exp([R_0-R_i]/b)^3$, using R_0 and b values from [2].[4]

Bond	n	R(Å)	BV	Bond	n	R(Å)	BV	Bond	n	R(Å)	BV
**V1-O1**	1	1.6544	1.452	**V1A-O5A**	1	1.738	1.170	**V1B-O1B**	1	1.679	1.362
**V1-O5**	1	1.751	1.132	**V1A-O1A**	1	1.667	1.405	**V1B-O5B**	1	1.73	1.195
**V1-O3**	2	1.9031	1.529	**V1A-O3A**	1	1.9041	0.763	**V1B-O3B**	1	1.899	0.773
**V1-O5**	1	2.3292	0.255	**V1A-O5A**	1	2.346	0.244	**V1B-O3B**	1	1.899	0.773
**V1-O4**	1	2.0725	0.494	**V1A-O3B**	1	1.912	0.747	**V1B-O5B**	1	2.302	0.274
**Sum**			**4.862**	**V1A-O4A**	1	2.054	0.518	**V1B-O4B**	1	2.082	0.482
				**Sum**			**4.848**	**Sum**			**4.858**
**V2-O3**	1	1.961	0.659	**V2A-O3B**	1	1.947	0.683	**V2B-O3A**	1	1.96	0.660
**V2-O4**	2	1.9232	1.452	**V2A-O4A**	1	1.919	0.734	**V2B-O4A**	2	1.935	1.409
**V2-O2**	1	1.6879	1.332	**V2A-O4A**	1	2.289	0.283	**V2B-O2B**	1	1.694	1.311
**V2-O4**	1	2.3152	0.264	**V2A-O2A**	1	1.698	1.297	**V2B-O4B**	1	2.262	0.303
**V2-O5**	1	1.8913	0.788	**V2A-O4B**	1	1.925	0.723	**V2B-O5B**	1	1.917	0.738
**Sum**			**4.495**	**V2A-O5A**	1	1.898	0.775	**Sum**			**4.421**
				**Sum**			**4.495**				
**Cu1-O2**	2	1.996	0.615	**Cu1A-O2A**	2	2.000	0.608	**Cu1B-O2B**	2	2.047	0.528
**Cu1-O1**	4	2.359	0.416	**Cu1A-O1B**	2	2.339	0.221	**Cu1B-O1A**	4	2.426	0.341
**Sum**			**1.031**	**Cu1A-O1A**	2	2.314	0.238	**Sum**			**0.869**
				**Sum**			**1.067**				
**Cu2-O1**	2	2.4493	0.159	**Cu2A-O1B**	1	2.538	0.061	**Cu2B-O1A**	2	2.402	0.183
**Cu2-O2**	2	1.943	0.721	**Cu2A-O2B**	2	1.925	0.760	**Cu2B-O2A**	2	1.996	0.615
**Sum**			**0.880**	**Cu2A-O1A**	1	2.402	0.092	**Sum**			**0.798**
				**Sum**			**0.913**				

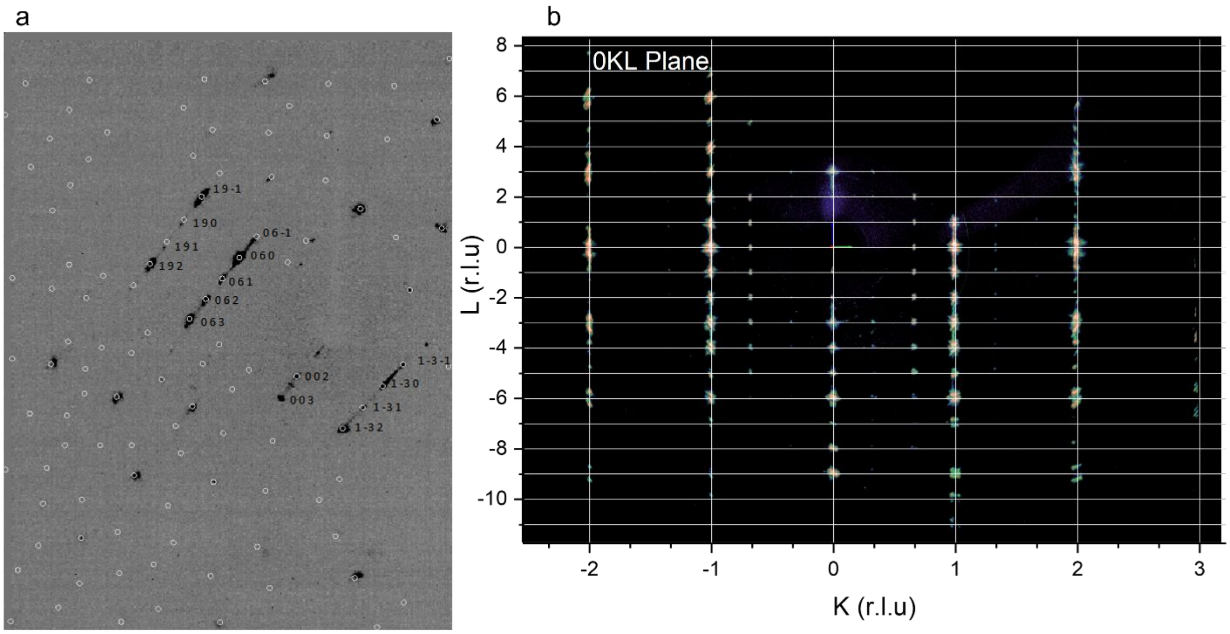

Figure S7. Crystallographic Evidence of Long-Range Positional Correlation and Structural Modulations in ε-$Cu_{0.9}V_2O_5$ Single Crystals at Low Temperature. (a) Diffractometer detector image showing spot asymmetry and significant diffuse scattering between diffraction spots along *c** for a single crystal at 110 K. Reflection indices are provided in the 1×3×1 supercell basis. (b) A slice of reciprocal space showing scattering intensity in the 0KL plane (integrated from -0.5KL to 0.5KL). Diffuse scattering intensity along L is readily visible, as well as spot splitting along both K and L directions, increasing in separation with increasing K. Indeed, by K=3, 03L and 03(L+1) reflections have split enough to nearly coincide at 03(L+0.5). The presence of diffuse scattering suggests positional correlation between Cu-ions in the *c** direction[5] (from Cu layer to Cu layer), whereas the spot splitting suggests that this correlation is transmitted through the intervening V_2O_5 sub-lattice layers by structural distortions (possibly accompanying electron localization) whose periodicity is incommensurate with the lattice structure.[6]

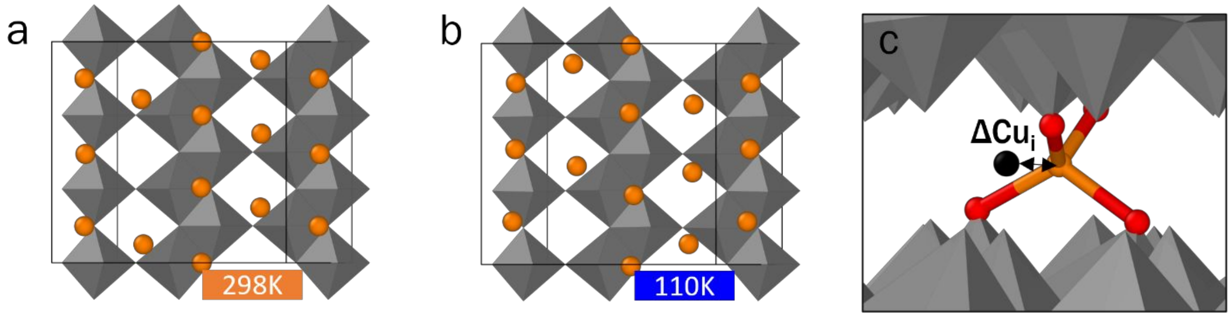

Figure S8. Molecular Dynamics Simulations of Inter-Site Cu-ion Migration

(a,b) Ground-state ε-$Cu_{0.9}V_2O_5$ calculated structures at 298 K and 110 K, respectively, used as starting points for AIMD simulations. (c) Schematic illustration of how copper-ion displacement from the nearest crystallographic site is quantified. Summation of this value over all copper-ions in the simulation cell gives the cumulative Cu-ion displacement depicted in Figure 4c of the main text.

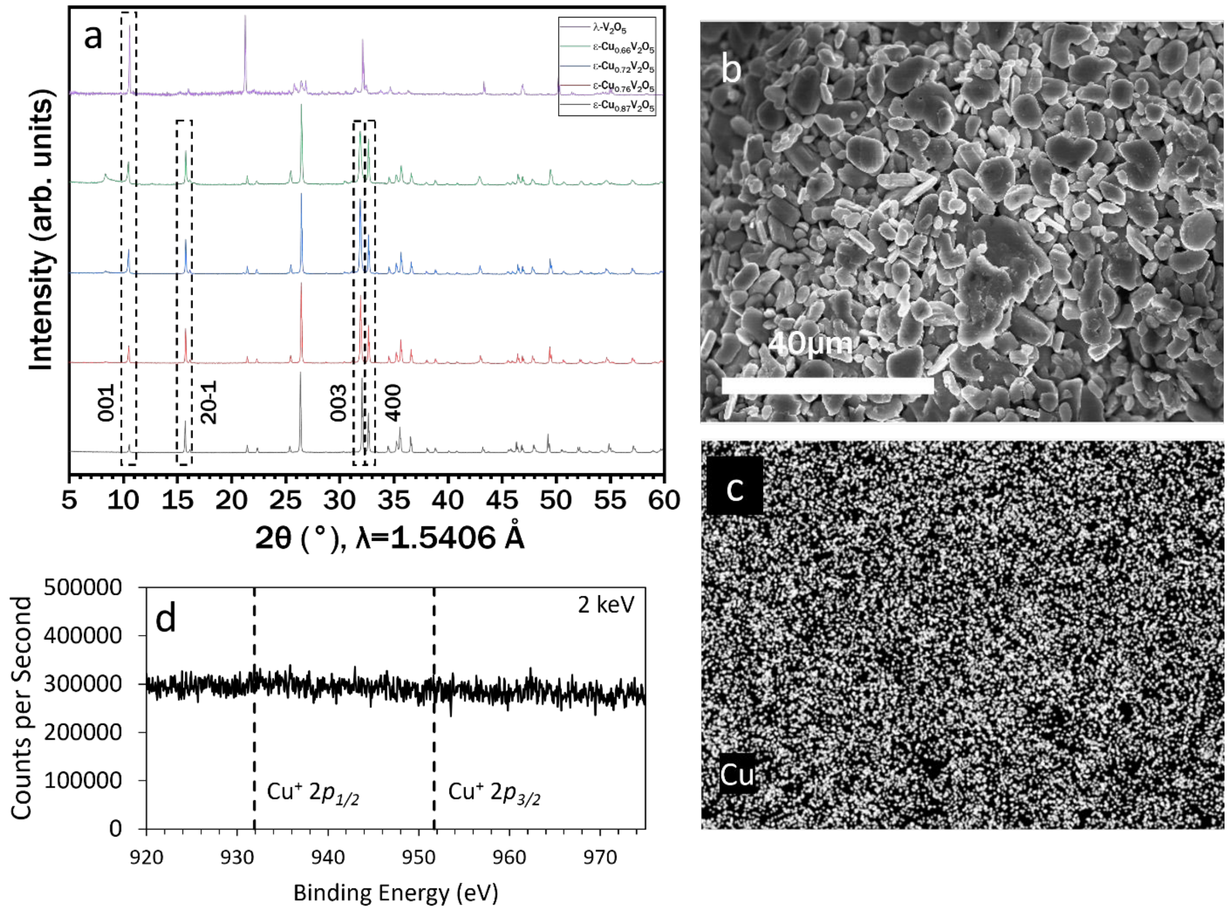

Figure S9. Characterization of As-Prepared and De-intercalated ε-$Cu_{0.9}V_2O_5$ Powders.

(a) Powder X-ray Diffraction patterns of as-prepared (black), and de-intercalated ε-$Cu_{0.9}V_2O_5$ powders with Cu stoichiometries noted in the legend. Nominal copper concentrations are as determined by EDS. Reflections exhibiting significant intensity change with copper removal are annotated. Additional reflections in the entirely deintercalated λ-V_2O_5 pattern are ascribed to formation of an additional layer stacking sequence in the absence of interlayer pillaring ions. (b,c) SEM image and corresponding EDS Cu composition map of ε-$Cu_{0.66}V_2O_5$ particles illustrating the homogeneous distribution of remaining copper. (d) A HAXPES spectrum of λ-V_2O_5 shows signal at binding energies expected for Cu 2*p* core levels (vertical dashed lines), indicating nearly complete copper-ion removal.

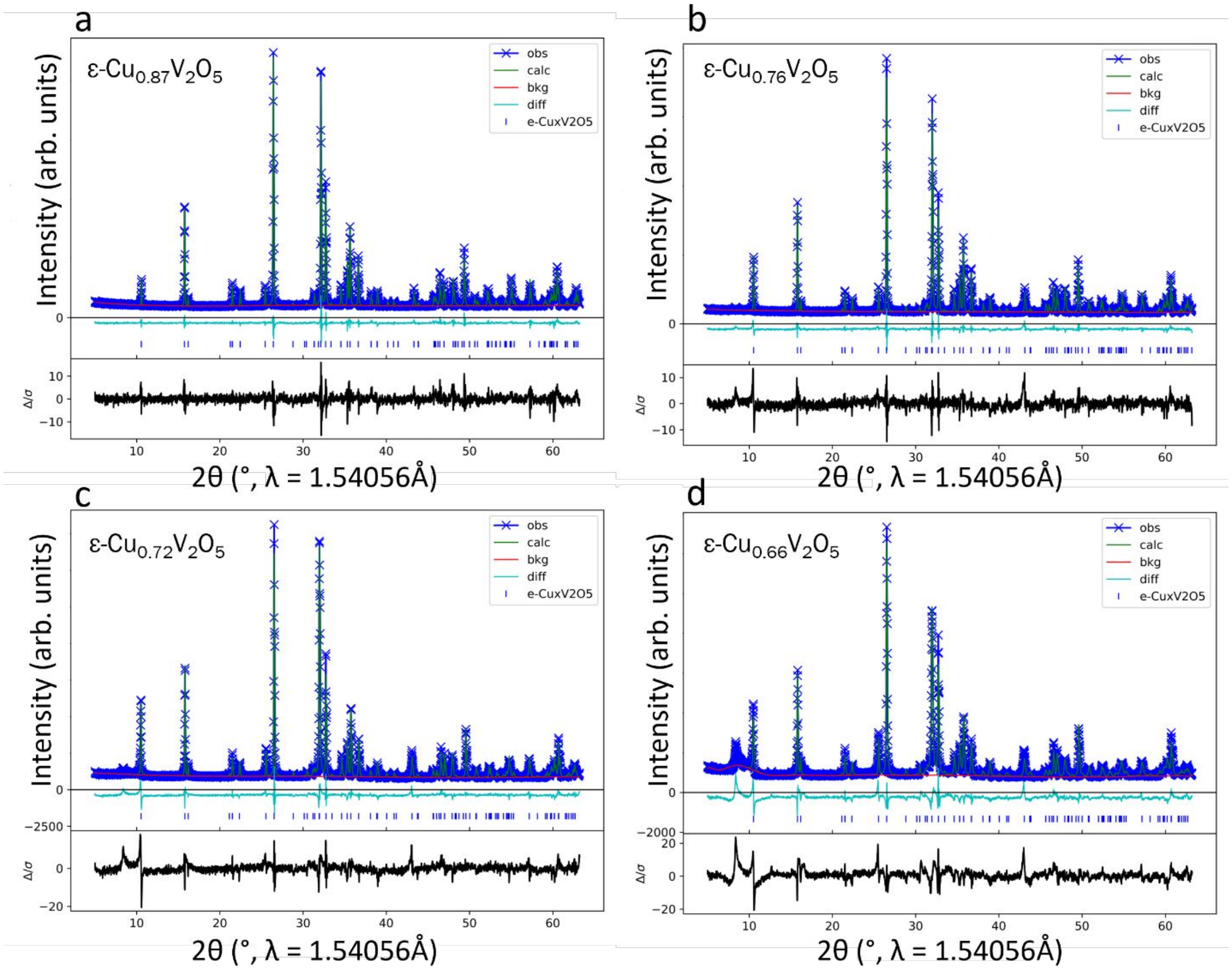

Figure S10. Rietveld refinement of As-Prepared and De-intercalated ε-$Cu_{0.9}V_2O_5$ Structure Models against powder X-ray diffraction data.

Rietveld refinement of the X-ray diffraction patterns of ε-$Cu_xV_2O_5$ for x = (a) 0.87 (b) 0.76 (c) 0.72 (d) 0.66. The data are shown as blue cross marks, and the result of the refinement is plotted as a solid green line. The background is shown as solid red line and the difference (observed—calculated) is shown as solid teal line. The vertical blue bars show the Bragg reflection positions for the refined structural models.

Table S6. Selected Rietveld refinement parameters pertaining to Figure S10.

	**ε-$Cu_{0.87}V_2O_5$**	**ε-$Cu_{0.76}V_2O_5$**	**ε-$Cu_{0.72}V_2O_5$**	**ε-$Cu_{0.66}V_2O_5$**
***a* (Å)**	11.7489(6)	11.729(8)	11.7241(11)	11.719(3)
***b* (Å)**	3.68802(4)	3.67720(5)	3.67442(7)	3.6721(2)
***c* (Å)**	8.9680(4)	8.9958(5)	8.9992(7)	8.998(2)
**β (°)**	111.5182(11)	111.2815(15)	111.2241(21)	111.173(5)
**V (Å^3)**	361.50(1)	361.541(12)	361.38(2)	361.09(4)
**Cu1 occupancy**	0.941(6)	0.870(7)	0.837(8)	0.785(14)
**Cu2 occupancy**	0.421(4)	0.369(4)	0.343(5)	0.362(11)
**Cu per V_2O_5**	0.892(7)	0.804(8)	0.762(9)	0.755(18)

Although ε-$Cu_xV_2O_5$ formally adopts *C2/m* symmetry, all powder samples were refined using a reduced-symmetry *Cm* unit cell containing twice as many V and O atoms to better model local disorder on the V_2O_5 lattice caused by partial copper removal. Prominent trends in *a*, *b*, and β are consistent across stoichiometries. Nominal copper stoichiometries are derived from energy-dispersive X-ray spectroscopy results.

	Area of Interest	Stoichiometric Coefficient per 2V					
		**V**	**O**	**Cu**	**Al**	**Si**	**C**
ε-$Cu_{0.87}V_2O_5$	**1**	2	3.014	0.870	0.153		0.716
	**2**	2	2.937	0.866	0.147		0.713
	**3**	2	3.240	0.875	0.155		0.922
	**Average**	**2**	**3.064**	**0.870**	**0.152**		**0.784**
	**St. Dev.**	0	0.129	0.004	0.004		0.098
ε-$Cu_{0.76}V_2O_5$	**1**	2	3.372	0.760	0.415	0.000	1.157
	**2**	2	3.823	0.766	0.262	0.000	1.965
	**3**	2	3.224	0.755	0.236	0.010	1.817
	**Average**	**2**	**3.473**	**0.760**	**0.304**	**0.003**	**1.646**
	**St. Dev.**	0	0.255	0.004	0.079	0.005	0.351
ε-$Cu_{0.72}V_2O_5$	**1**	2	2.725	0.720	0.325	0.008	0.727
	**2**	2	2.649	0.716	0.299	0.000	0.902
	**3**	2	3.336	0.722	0.290	0.010	1.277
	**Average**	**2**	**2.903**	**0.720**	**0.304**	**0.006**	**0.968**
	**St. Dev.**	0	0.308	0.002	0.015	0.004	0.230
ε-$Cu_{0.66}V_2O_5$	**1**	2	4.012	0.650	0.374	0.008	1.371
	**2**	2	3.145	0.665	0.277	0.000	0.504
	**3**	2	3.024	0.650	0.329	0.012	1.213
	**Average**	**2**	**3.393**	**0.655**	**0.326**	**0.007**	**1.029**
	**St. Dev.**	0	0.440	0.007	0.040	0.005	0.377
λ-V_2O_5	**1**	2	2.840	0.009	0.069		0.796
	**2**	2	3.116	0.007	0.143		0.423
	**Average**	**2**	**2.978**	**0.008**	**0.106**		**0.609**
	**St. Dev.**	0	0.138	0.001	0.037		0.186

Table S7. Quantitative Energy-Dispersive X-ray Spectroscopy (EDX) Results for Pristine and De-intercalated ε-$Cu_xV_2O_5$ Powders. Reported copper concentrations are averages across several areas of interest. The presence of Si is attributed to residual silica ampoule material introduced during synthesis. The aluminum and carbon signals originate from the sample mount and conductive tape, respectively. λ-V_2O_5 is observed to contain < 1% of its original copper content.

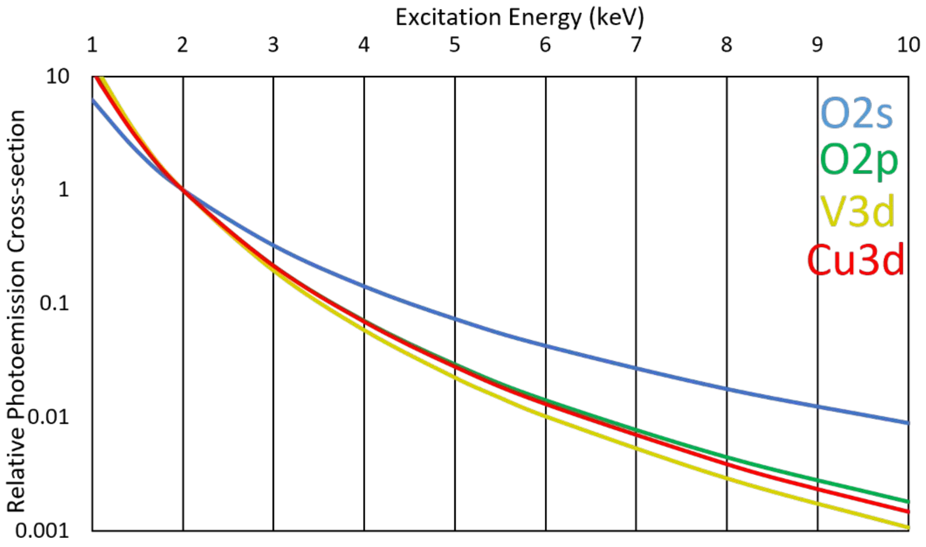

Figure S11. Photoemission Cross-section Decay Profiles for Relevant Atomic Orbitals

Photoemission cross-sections of atomic orbitals normalized to their value at 2 keV excitation energy.[7,8] By comparing spectra collected at 2 keV to those at 5 keV, orbital contributions to spectral features can be differentiated.

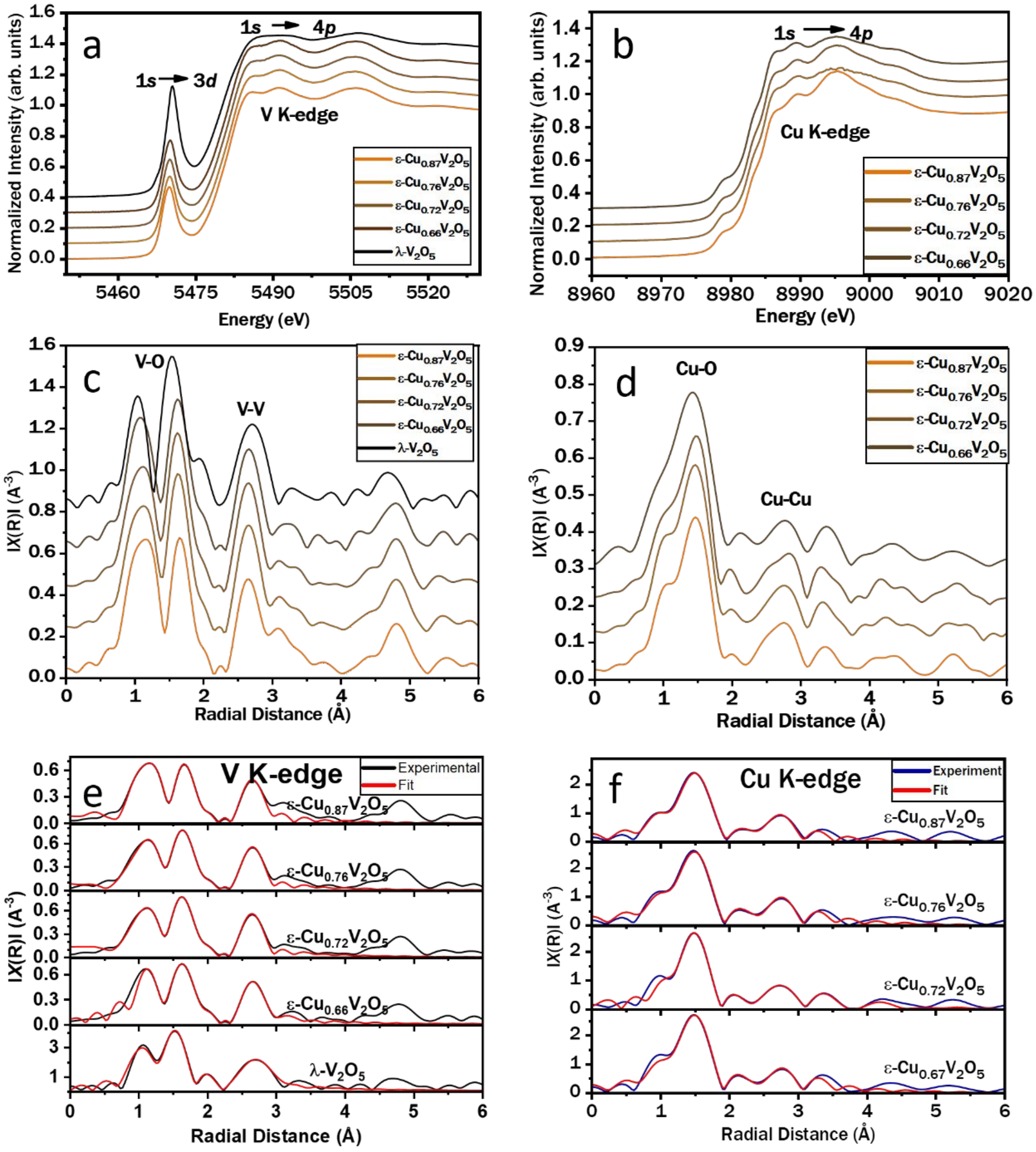

Figure S12. V and Cu K-edge XANES and EXAFS Fitting Results for ε-$Cu_{0.9}V_2O_5$ and partially deintercalated ε-$Cu_xV_2O_5$ powders. (a,b) V (a) and Cu (b) K-edge XANES spectra of pristine and de-intercalated powders. Although V1*s* → V3*d* transitions are formally forbidden by dipole selection rules (angular momentum = ±1), off-centering of V ions hybridizes 3*d* and 4*p* states and results in the prominent pre-edge absorption feature at 5470 eV in (a). (c,d) k^3-weighted Fourier transforms of the V (c) and Cu (d) K-edge EXAFS spectra of partially and fully de-intercalated samples. (e,f) Fitted R-space V (e) and Cu (f) K-edge EXAFS spectra of partially and fully de-intercalated samples. In (c), the downward shift of overall intensity of the first prominent peak (around 1 Å) results from the increasing V off-centering and consequent vanadyl bond length contraction with vanadium oxidation. Cu de-intercalation leads to broadening of peaks associated with the first coordination shell of Cu (d) as decreased Cu concentration reduces the strength of positional correlation.

Table S8. Tabulated V K-edge EXAFS Fitting Parameters. Atomic positions obtained from high-resolution single-crystal X-ray diffraction (Tables S2-S5) were used to fit the EXAFS data. Fitting parameters corresponding to the spectra in Figure S7(a-b). Of particular note is the trend of decreased vanadyl (V=O) bond length with copper removal, and the accompanying increase in V-O_{trans} bond length, both due to increased vanadium off-centering.

Sample	Path	N	R(Å)	σ^2(Å)^2	S_0^2	E_0 (eV)	R-factor (%)
**λ-V_2O_5**	V=O	1	1.400	0.003	1.0	3.002 ± 0.068	0.5
	V—O_{trans}	1	2.699				
	V—V	2	3.123				
**ε-$Cu_{0.87}V_2O_5$**	V=O	1	1.637	0.001	1.0	1.012 ± 0.055	0.4
	V—O_{trans}	1	2.128				
	V—V	2	2.955				
**ε-$Cu_{0.76}V_2O_5$**	V=O	1	1.572	0.014	1.0	1.420 ± 0.126	0.7
	V—O_{trans}	1	2.261				
	V—V	2	2.956				
**ε-$Cu_{0.72}V_2O_5$**	V=O	1	1.567	0.004	1.0	1.741 ± 0.523	0.3
	V—O_{trans}	1	2.130				
	V—V	2	2.949				
**ε-$Cu_{0.67}V_2O_5$**	V=O	1	1.624	0.005	1.0	1.414 ± 0.717	0.1
	V—O_{trans}	1	2.291				
	V—V	2	2.996				

Table S9. Tabulated Cu K-edge EXAFS Fitting Parameters. Atomic positions obtained from high-resolution single-crystal X-ray diffraction (Tables S2-S5) were used to fit the EXAFS data. Fitting parameters corresponding to the spectra in Supporting Figure S7(c-d). The fit reveals two distinct Cu coordination with no discernible trend in the Cu—O bond length upon Cu removal, consistent with reported Cu intercalated aerogel and xerogel V_2O_5.

Sample	Path	N	R(Å)	σ^2(Å)^2	S_0^2	E_0 (eV)	R-factor (%)
**ε-$Cu_{0.87}V_2O_5$**	Cu—O	4	1.980	0.018	1.0	1.044 ± 0.063	0.5
	Cu—O	2	2.308				
	Cu—Cu	4	2.744				
**ε-$Cu_{0.76}V_2O_5$**	Cu—O	4	1.967	0.020	1.0	1.230 ± 0.252	0.5
	Cu—O	2	2.307				
	Cu—Cu	4	2.534				
**ε-$Cu_{0.72}V_2O_5$**	Cu—O	4	1.982	0.004	1.0	1.342 ± 0.212	0.2
	Cu—O	2	2.305				
	Cu—Cu	4	2.523				
**ε-$Cu_{0.67}V_2O_5$**	Cu—O	4	1.971	0.001	1.0	1.123 ± 0.221	0.3
	Cu—O	2	2.302				
	Cu—Cu	4	2.541				

EXAFS Fitting Results and Discussion

The first two peaks below 2 Å in the Fourier-transformed V K-edge EXAFS data (**Fig S12c)** can be assigned to the first V−O coordination shell, followed by an additional 3 peaks extending to 4 Å which include V−V and V−O single scattering paths along with various multiple scattering paths contributing the amplitude to these outer shell peaks. Focusing on the first coordination shell, a model considering one short V−O bond, and one long V−O_{trans} bond was utilized to analyze the EXAFS spectra and compare the local V environments in λ-V_2O_5 and ε-$Cu_xV_2O_5$. In the case of λ-V_2O_5, the fitting result in **Figure S12e** show that VO_6 octahedra have one short V−O bond ≈1.40 Å and one long bond ≈2.70 Å (**Table S7**) at the opposite position. However, after Cu intercalation, the fitting results show an expansion of the vanadyl bond ((1.57 to 1.62) Å) and concurrent contraction of the trans bond ((2.29 to 2.13) Å) in ε-$Cu_xV_2O_5$, suggesting that VO_6 octahedra become more symmetrical upon Cu intercalation.

In contrast, the Cu EXAFS of ε-$Cu_xV_2O_5$ (**Figure S12d**) reveals that the peaks at 1.3 Å and (2.0 to 2.7) Å can be ascribed to the first Cu−O coordination shell and (Cu-O, Cu-Cu) single/multiple scattering paths respectively. The fitting results (**Figure S12f**) show that the Cu-O bond lengths ((1.967 to 1.983 Å; **Table S8**) in ε-$Cu_xV_2O_5$ is similar to those reported for Cu intercalated aerogel ((1.954 to 1.970) Å) and xerogel ((1.953 to 1.956) Å) V_2O_5,[9] as well as β'-$Cu_xV_2O_5$ ((1.9 to 1.95) Å) [10] where Cu is 4-fold coordinated by almost coplanar oxygens. In addition, the fit result yielded a Cu-O bond length of (2.321 to 2.415 Å), which again is consistent with the Cu-O_{ax} (2.14 to 2.32) Å in $[Cu(H_2O)_6](ClO_4)_2$ albeit a slight elongation, where Cu is 6-fold coordinated.[11] These observations corroborate that Cu exhibits a preference for both square planar and octahedral sites within this compound.

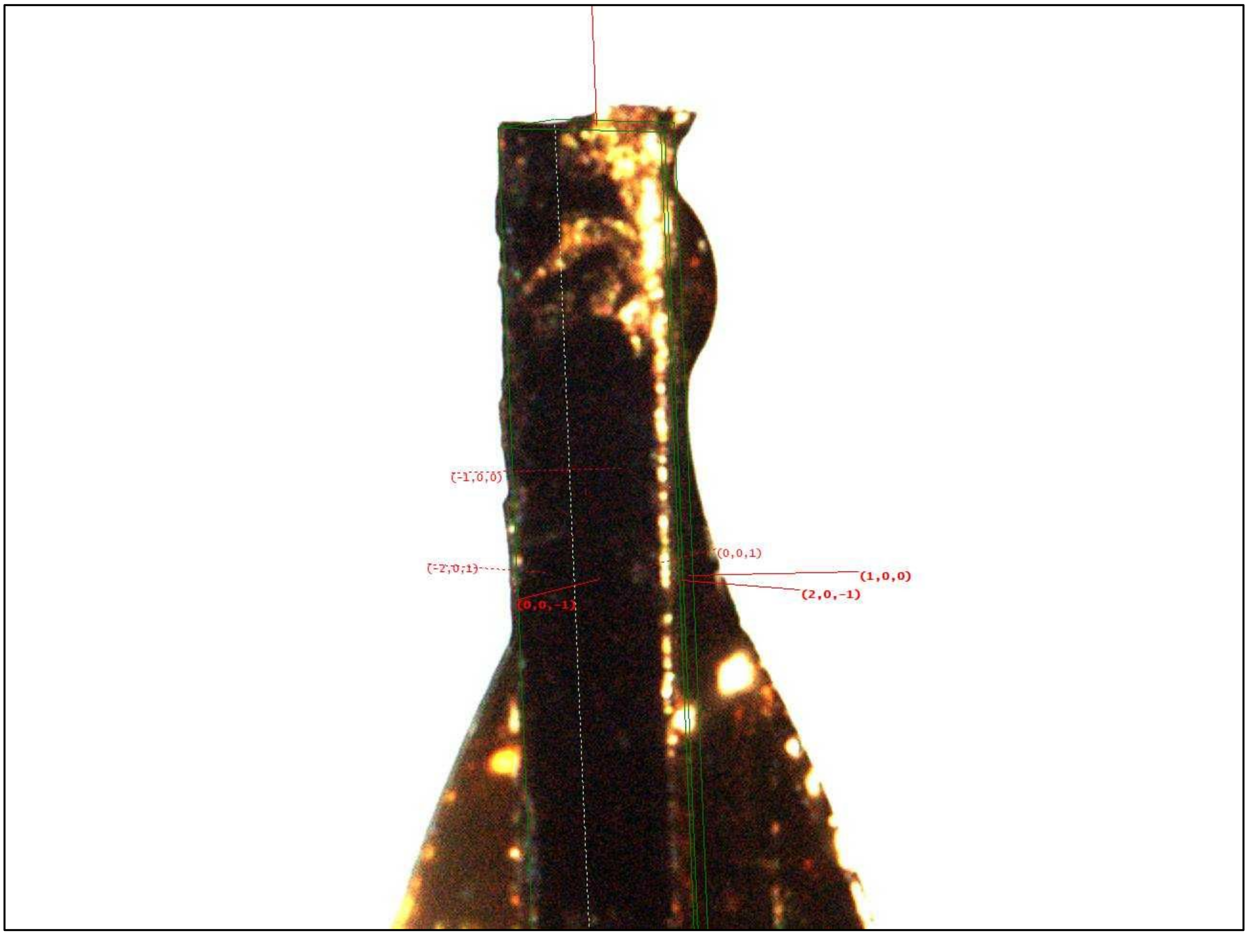

Figure S13. Face indexing of single crystals. A large ε-$Cu_{0.9}V_2O_5$ single crystal indexed during a diffraction experiment. Miller indices of visible and hidden faces indicated by solid and dashed red lines, respectively.

Supporting References:

(1) Agbeworvi, G.; Zaheer, W.; Handy, J. V.; Andrews, J. L.; Perez-Beltran, S.; Jaye, C.; Weiland, C.; Fischer, D. A.; Balbuena, P. B.; Banerjee, S. Toggling Stereochemical Activity through Interstitial Positioning of Cations between 2D V_2O_5 Double Layers. *Chem. Mater.* **2023**, *35* (17), 7175–7188. https://doi.org/10.1021/acs.chemmater.3c01463.

(2) Brown, T. D.; Kumar, S.; Williams, R. S. Physics-Based Compact Modeling of Electro-Thermal Memristors: Negative Differential Resistance, Local Activity, and Non-Local Dynamical Bifurcations. *Appl. Phys. Rev.* **2022**, *9* (1), 011308. https://doi.org/10.1063/5.0070558.

(3) Brown, I. D.; Altermatt, D. Bond-Valence Parameters Obtained from a Systematic Analysis of the Inorganic Crystal Structure Database. *Acta Crystallogr. B* **1985**, *41* (4), 244–247. https://doi.org/10.1107/S0108768185002063.

(4) Gagné, O. C.; Hawthorne, F. C. Comprehensive Derivation of Bond-Valence Parameters for Ion Pairs Involving Oxygen. *Acta Crystallogr. Sect. B Struct. Sci. Cryst. Eng. Mater.* **2015**, *71* (5), 562–578. https://doi.org/10.1107/S2052520615016297.

(5) Krogstad, M. J.; Rosenkranz, S.; Wozniak, J. M.; Jennings, G.; Ruff, J. P. C.; Vaughey, J. T.; Osborn, R. Reciprocal Space Imaging of Ionic Correlations in Intercalation Compounds. *Nat. Mater.* **2020**, *19* (1), 63–68. https://doi.org/10.1038/s41563-019-0500-7.

(6) Van Smaalen, S. Incommensurate Crystal Structures. *Crystallogr. Rev.* **1995**, *4* (2), 79–202. https://doi.org/10.1080/08893119508039920.

(7) Kalha, C.; Fernando, N.; Regoutz, A. Digitisation of Scofield Photoionisation Cross Section Tabulated Data. *Figshare* **2020**. https://doi.org/10.6084/m9.figshare.12967079.v1.

(8) Scofield, J. H. *Theoretical Photoionization Cross Sections from 1 to 1500 keV.*; Divison of Technical Information Extension: U.S. Atomic Energy Commission, 1973. https://doi.org/10.2172/4545040.

(9) Giorgetti, M.; Berrettoni, M. Doped V2O5-Based Cathode Materials: Where Does the Doping Metal Go? An X-Ray Absorption Spectroscopy Study. *Chem. Mater.* **2007**, *19* (24), 5991–6000. https://doi.org/10.1021/cm701910c.

(10) Parija, A.; Handy, J. V.; Andrews, J. L.; Wu, J.; Wangoh, L.; Singh, S.; Jozwiak, C.; Bostwick, A.; Rotenberg, E.; Yang, W.; Fakra, S. C.; Al-Hashimi, M.; Sambandamurthy, G.; Piper, L. F. J.; Williams, R. S.; Prendergast, D.; Banerjee, S. Metal-Insulator Transitions in β′-$Cu_xV_2O_5$ Mediated by Polaron Oscillation and Cation Shuttling. *Matter* **2020**, *2* (5), 1166–1186. https://doi.org/10.1016/j.matt.2020.01.027.

(11) Persson, I.; Lundberg, D.; Bajnóczi, É. G.; Klementiev, K.; Just, J.; Sigfridsson Clauss, K. G. V. EXAFS Study on the Coordination Chemistry of the Solvated Copper(II) Ion in a Series of Oxygen Donor Solvents. *Inorg. Chem.* **2020**, *59* (14), 9538–9550. https://doi.org/10.1021/acs.inorgchem.0c00403.
